# Li_2_ZnTi_3_O_8_/graphene nanocomposite as a high-performance anode material for lithium-ion batteries†

**DOI:** 10.1039/c8ra05893h

**Published:** 2018-09-10

**Authors:** Song Wang, Lijuan Wang, Zhaohui Meng, Baomin Luo

**Affiliations:** School of Chemistry and Material Science, Liaoning Shihua University Fushun 113001 Liaoning China lijuanw123@163.com +86-24-56861709 +86-24-56861711; College of Chemistry and Pharmaceutical Engineering, Nanyang Normal University Nanyang 473061 Henan China

## Abstract

An Li_2_ZnTi_3_O_8_/graphene (LZTO/G) anode is successfully synthesized by a two-step reaction. The results show that LZTO particles can be well dispersed into the graphene conductive network. The conductive structure greatly improves the electrochemical performance of LZTO/G. When cycled for 400 cycles, 76.4% of the capacity for the 2nd cycle is maintained at 1 A g^−1^. Also, 174.8 and 156.5 mA h g^−1^ are still delivered at the 100th cycle for 5 and 6 A g^−1^, respectively. The excellent cyclic performance and the large specific capacities at high current densities are due to the good conductive network of the LZTO active particles, large pore volume, small particle size, low charge-transfer resistance and high lithium diffusion coefficient.

## Abstract 2

The structure of G modifying LZTO is beneficial for the diffusion of Li^+^ ions and transportation of electrons.

## Graphical abstract

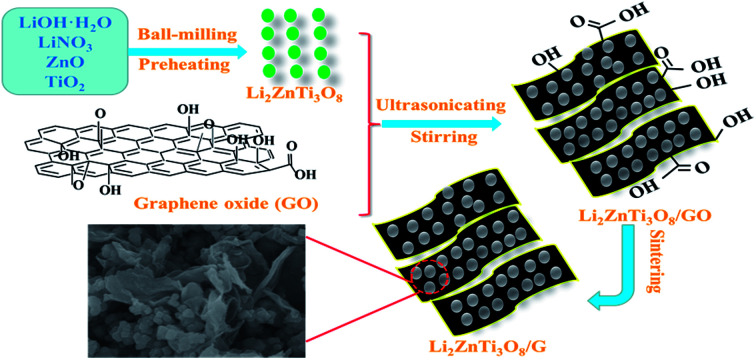

## Introduction

Spinel Li_2_ZnTi_3_O_8_ (LZTO) is highly competitive as an anode material for lithium-ion batteries (LIBs) due to its environment-friendly raw materials, good safety and simple synthetic process.^1–11^ The insertion/deinsertion reaction for Li^+^ ions in LZTO can be expressed as follows:

1 Li_2_ZnTi_3_O_8_ + 3Li^+^ + 3e^−^ ↔ Li_5_ZnTi_3_O_8_

As seen in eqn (1), Ti^4+^ can be reduced to Ti^3+^ when discharged to 0 V in LZTO with relatively large theoretical capacity of 227 mA h g^−1^. However, its electronic conductivity and rate capability need to be greatly improved.

The preparation of conductive carbon coating on nanosized LZTO is considered as an effective approach to improve its electrochemical performance.^12–16^ The conductive carbon coating can enhance the electronic conductivity of LZTO, and nano-sized particles improve the diffusion of Li^+^ ions. Recently, graphene (G), an ideal carbon source with high electronic conductivity, has been widely used to modify cathode and anode materials for LIBs to improve their electrochemical performance.^17–23^ However, to the best of our knowledge, no research on the synthesis of nanosized LZTO/G using G as the conductive carbon has been reported.

In this paper, we report for the first time, the preparation of LZTO/G nanocomposite with excellent high rate capability and cyclic performance. LZTO nanoparticles are enwrapped in a conductive network of G layers. The existence of G greatly improves the electrochemical performance of LZTO.

## Experimental

### Synthesis of graphene oxide (GO) sheets

GO sheets were prepared *via* a modified Hummers method^24,25^ and our previously reported method.^26^ The synthesis process is shown in the ESI.†

### Synthesis of Li_2_ZnTi_3_O_8_/graphene (LZTO/G)

The LZTO/G anode was synthesized by the molten-salt method similar to our previous method.^15^ TiO_2_ (anatase, A.R.), LiOH·H_2_O (A.R.), LiNO_3_ (A.R.) and ZnO (A.R.) were ball-milled for 5 h with Li/Zn/Ti = 2.4 : 1 : 3. The molar ratio of LiOH·H_2_O to LiNO_3_ was 0.38 : 0.62. The mixture was dried at 80 °C for 12 h in air, pre-heated at 250 °C for 3 h and then at 600 °C for 4 h in air, mixed with GO sheets by ultrasonically treating for 2 h, stirred for 24 h using ethanol as the dispersing medium, dried at 80 °C in air and finally sintered at 700 °C for 3 h in N_2_/H_2_ (v/v = 93 : 7). The obtained material was denoted as LZTO/G. For comparison, LZTO was also fabricated by the same method without GO sheets.

### Physical and electrochemical performance measurements

The related physical and electrochemical performance measurements are shown in the ESI† in detail.

## Results and discussion

**Fig. 1:**
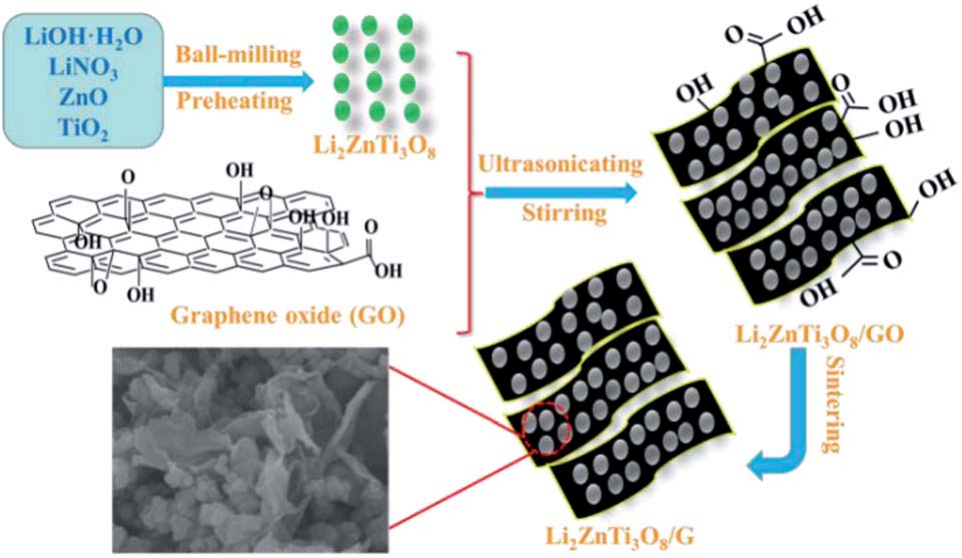
Schematic illustration for the synthesis process of the LZTO/G nanocomposite.

Fig. 1 exhibits a schematic of the two-step reaction for the synthesis of the LZTO/G nanocomposite. In the first step, LZTO is prepared by preheating the precursors of LiOH·H_2_O, LiNO_3_, ZnO and TiO_2_ at 250 °C for 3 h and then at 600 °C for 4 h in air (Fig. S1a and b†). In the second step, the mixture of LZTO and GO is ultrasonicated, stirred and sintered in N_2_/H_2_. The LZTO nanoparticles can be completely absorbed on and combined with GO sheets due to the existence of carboxyl, hydroxyl and epoxy groups^27^ on the surfaces of GO sheets (Fig. S1c†), which can subsequently be converted to G at a high temperature in reductive atmosphere. Then, a conductive G network enwrapping the LZTO particles is formed. The structure is beneficial to the insertion and deinsertion of Li^+^ ions. In addition, G has outstanding electronic behavior and makes the electronic conductivity of LZTO/G reach 0.3767 S cm^−1^, which is greatly larger than that of LZTO (7.7 × 10^−6^ S cm^−1^) (Table S1†).

The XRD patterns of GO and G are shown in Fig. S2a.† The (002) reflection peak of GO (2*θ* = 8.9°) shifts to a higher value (2*θ* = 26.2°) in G. Based on the Bragg's equation 2*d* sin *θ* = *nλ*, where *d* is the interlayer spacing, *θ* is the diffraction angle, *n* is the diffraction order, and *λ* is the wavelength of Cu Kα, the interlayer spacings of GO and G are determined as 0.982 and 0.339 nm, respectively. The expansion of *d* indicates the formation of oxygen-containing groups between the GO layers. The decrease in *d* verifies the removal of oxygen functional groups in G.

The Raman spectra of GO and G are shown in Fig. S2b.† The spectrum displays two bands at 1357 cm^−1^ (D band, disorder-induced phonon mode) and 1605 cm^−1^ (G-band, graphite band) for each sample. The *I*_D_/*I*_G_ intensity ratio indicates the degree of disorder and can be used to confirm the reduction process. The *I*_D_/*I*_G_ ratio values are 0.945 and 0.988 for GO and G, respectively. It can be seen that the value increases after reduction, indicating that oxygen moieties are removed during the reduction process.^28^

The morphologies of GO and G are characterized by SEM and TEM (Fig. S2c–f†). GO displays typically rippled and crumpled surfaces (Fig. S2c†). G has sheet-like structures (Fig. S2d†). It can be seen from the TEM images (Fig. S2e and f†) that the multilayered GO sheets evolve into single or several layered G after reduction at 700 °C.

**Fig. 2:**
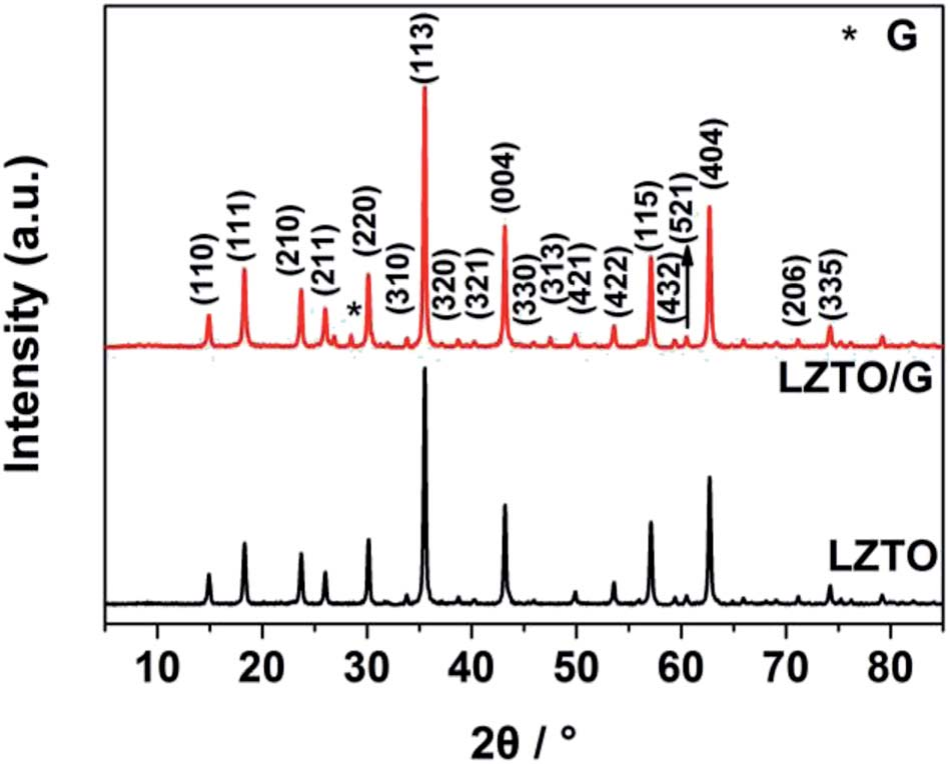
X-ray diffraction patterns of the LZTO and LZTO/G anode materials.

The XRD patterns of LZTO and LZTO/G anode materials are depicted in Fig. 2. For each sample, the diffraction peaks can be assigned to the cubic spinel structure of LZTO (JCPDS#44-1037). For the LZTO/G sample, the diffraction peak related to G is detected. The G content in LZTO/G composite is 8.67 wt% (Fig. S3†). XPS and Raman results further confirm that GO is reduced to G. (Fig. S4†). The lattice parameters are listed in Table S2.† Compared with LZTO, LZTO/G has larger lattice parameters, which may originate from the creation of oxygen vacancies due to the incomplete crystallinity of LZTO with the existence of G;^29,30^ this is suitable for rapid transportation of Li^+^ ions.^29^

**Fig. 3:**
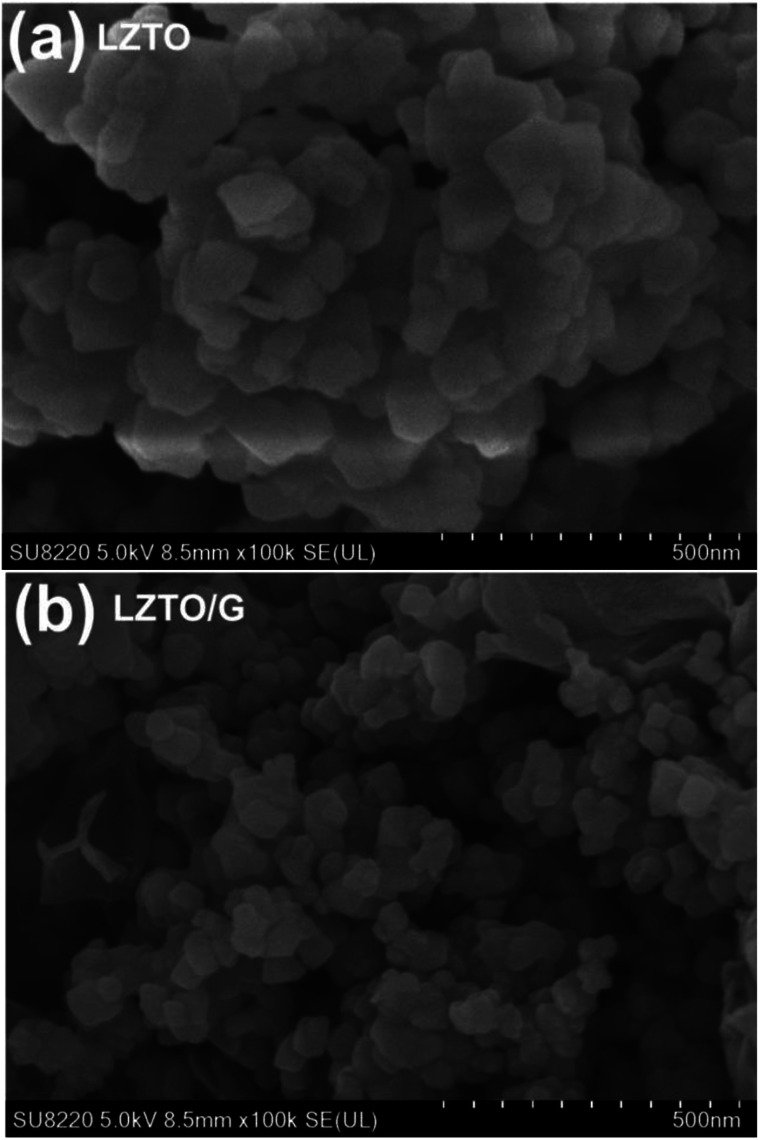
SEM images of (a) LZTO and (b) LZTO/G.

SEM images of the LZTO and LZTO/G anode materials are shown in Fig. 3. Compared with LZTO, the LZTO/G sample has a smaller particle size; it is looser as well as more porous due to the existence of G. The introduction of G greatly enhances the specific surface area and the total pore volume of LZTO (Fig. S5 and Table S3†). The large specific surface area and pore volume are beneficial for fast diffusion of Li^+^ ions.

**Fig. 4:**
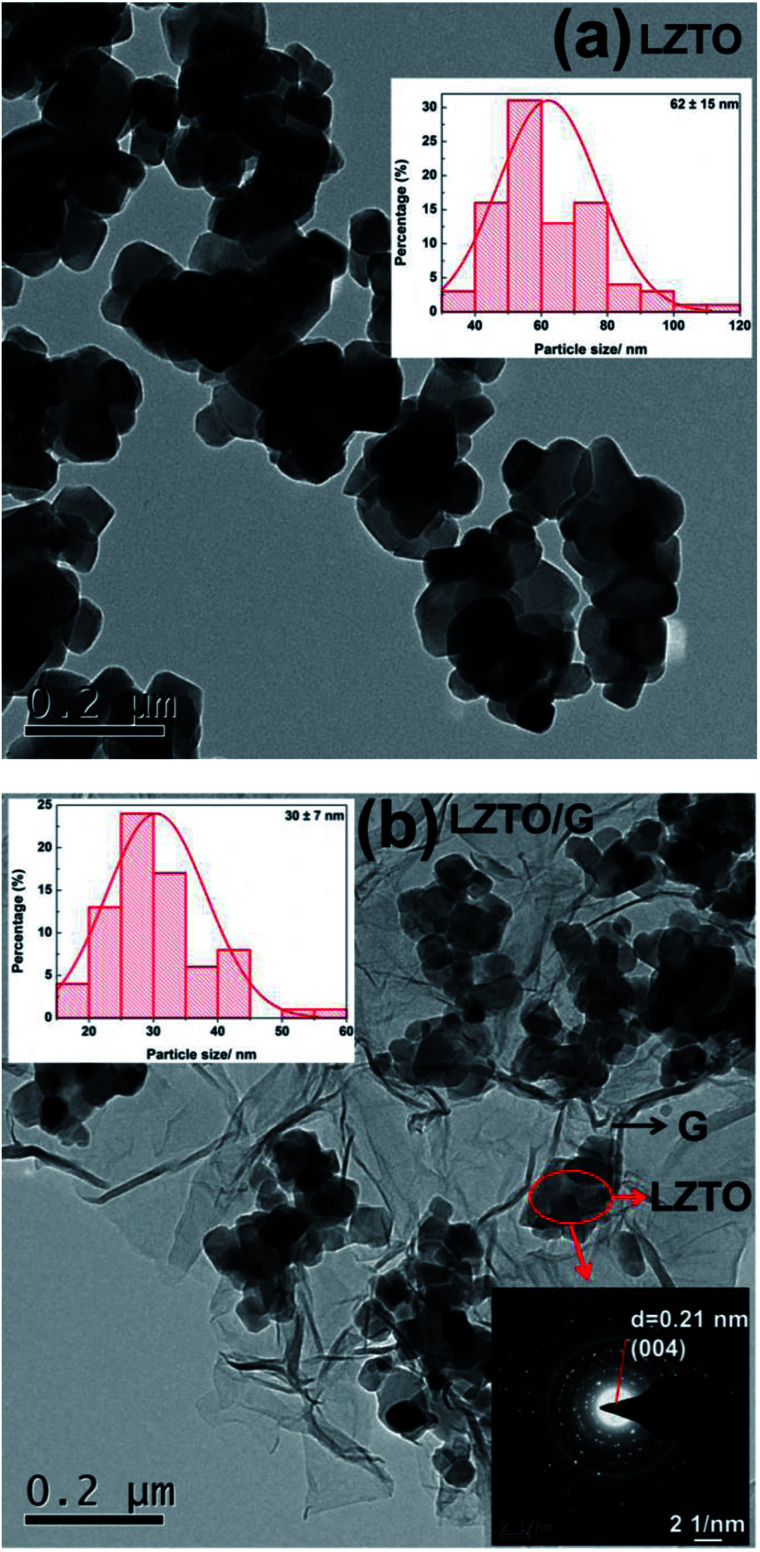
TEM images, histograms of particle size distribution (inset, top) and selected area electron diffraction (SAED) pattern (inset, bottom) of (a) LZTO and (b) LZTO/G.

TEM images of LZTO and LZTO/G are provided in Fig. 4. It is clear that there is a thin G layer coating on LZTO particles (Fig. 4b). The energy dispersive spectroscopy (EDS) results (Fig. S6b†) indicate that O, Ti, Zn and C exist in the LZTO/G composite. The elemental mappings (Fig. S6c–f†) suggest that the elements are uniformly dispersed.

Compared with LZTO, LZTO/G has a smaller particle size of 30 nm, further verifying that the existence of G can prohibit particle growth. Small particle size benefits the rate capability of LZTO/G. Selected-area electron diffraction (SAED) pattern shows that the LZTO particles are polycrystalline in the LZTO/G composite.

**Fig. 5:**
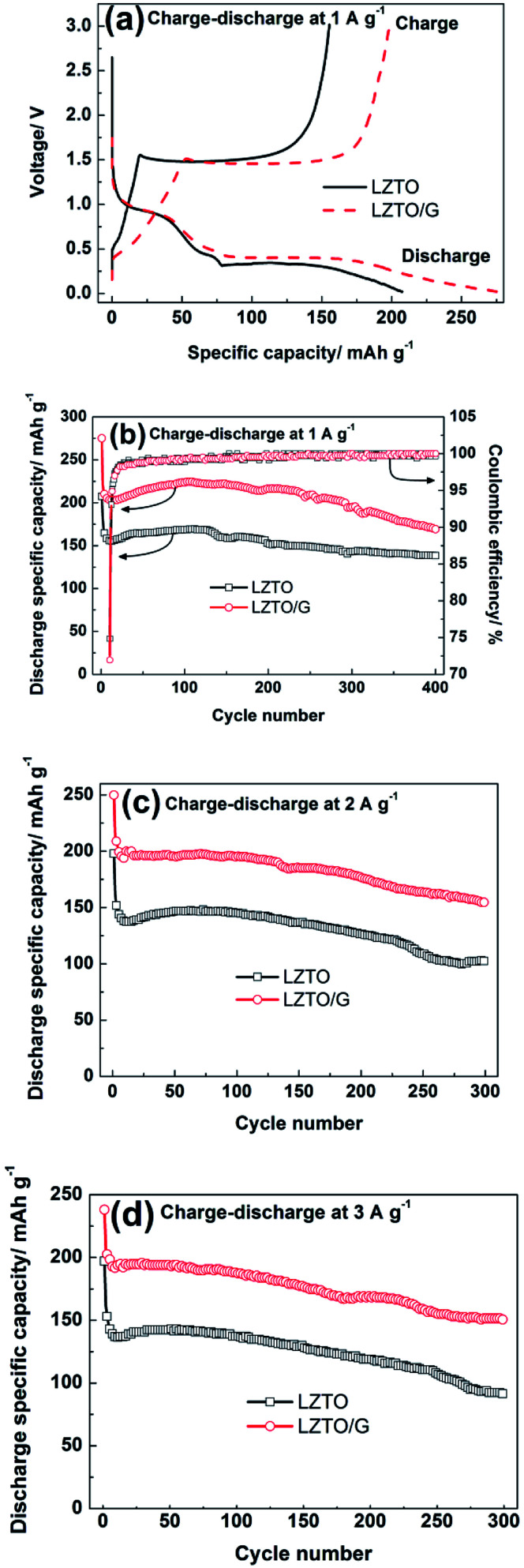
(a) Initial charge–discharge curves and (b) cyclic performances (inset, the corresponding columbic efficiency) of LZTO and LZTO/G at 1 A g^−1^ from 0.02 to 3.0 V (*vs.* Li/Li^+^); cyclic performances of LZTO and LZTO/G at (c) 2 A g^−1^ and (d) 3 A g^−1^ from 0.02 to 3.0 V (*vs.* Li/Li^+^).

Fig. 5a exhibits the initial charge–discharge curves of LZTO and LZTO/G at 1 A g^−1^ in the range of 0.02–3.0 V. For each sample, a charge plateau (1.48 V) and a corresponding discharge plateau (0.37 V) are observed, which indicate the typical electrochemical reaction of LZTO;^1–11^ this is further confirmed by the results of the CV technique (Fig. S7 and Table S4†). Compared with LZTO, LZTO/G has larger specific capacity of 275.0 mA h g^−1^ (Table 1) due to its larger specific surface area and the existence of G. However, the larger specific surface area of LZTO/G can also result in more side reactions and lower coulombic efficiency of 72.0% (Table 1). After several cycles, the coulombic efficiencies are close to 100% for the two samples, which indicates that large reversible capacities can be obtained (Fig. 5b). The cyclic performances of LZTO and LZTO/G at 1 A g^−1^ are shown in Fig. 5b. Also, 182.3 and 221.4 mA h g^−1^ are delivered at the 2nd cycle for LZTO and LZTO/G, respectively. When cycled for 400 cycles, 75.8% and 76.4% of the capacities for the 2nd cycle are maintained. When the current densities increase, LZTO/G still has better electrochemical performance. For instance, at 2 A g^−1^, 213.9 mA h g^−1^ is delivered at the 2nd cycle with the capacity retention of 72.3% at the 300th cycle (Fig. 5c and Table 2); at 3 A g^−1^, 150.7 mA h g^−1^ is obtained with the capacity retention of 72.7% at the 300th cycle (corresponding to that at the 2nd cycle) (Fig. 5d and Table 2). The large capacity or good cyclic performance of LZTO/G exceeds many previously reported values (Table S5†).

**Table 1:** Discharge capacities, coulombic efficiency and capacity retention of LZTO and LZTO/G at 1 A g^−1^

Samples	Specific capacity at the 1st cycle (mA h g^−1^)	Coulombic efficiency	Specific capacity at the 400th cycle (mA h g^−1^)	Capacity retention for the 2nd cycle
LZTO	207.5	74.9%	182.3	75.8%
LZTO/G	275.0	72.0%	221.4	76.4%

**Table 2:** Discharge capacities and capacity retention at the 300th cycle of LZTO and LZTO/G at 2 and 3 A g^−1^

Current density (A g^−1^)	LZTO, specific capacity at the 2nd cycle (mA h g^−1^)	Capacity retention for the 2nd cycle	LZTO/G, specific capacity at the 2nd cycle (mA h g^−1^)	Capacity retention for the 2nd cycle
2	162.4	63.1%	213.9	72.3%
3	91.5	56.4%	150.7	72.7%

**Fig. 6:**
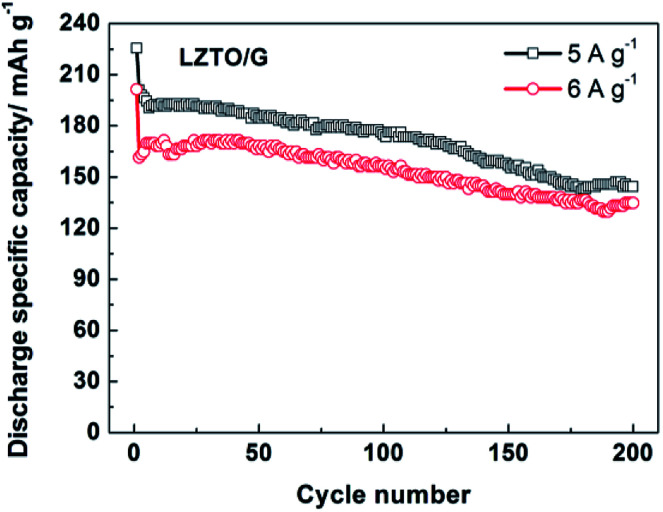
Cyclic performance of the LZTO/G electrode at high current densities of 5 and 6 A g^−1^ in the range of 0.02–3.0 V.

In addition, the electrode kinetics of LZTO and LZTO/G were investigated. Compared with LZTO, LZTO/G exhibited smaller charge transfer resistance of 187.6 Ω and higher lithium-ion diffusion coefficient of 3.65 × 10^−15^ cm^2^ s^−1^ (Fig. S8 and Table S6†), which indicated good rate capability of LZTO/G. Also, 174.8 and 156.5 mA h g^−1^ were still delivered at the 100th cycle for 5 and 6 A g^−1^, respectively (Fig. 6). The capacities were larger and the cyclic performance was better than many previously reported results (Table S5†).

To further clarify different electrochemical behaviors of LZTO and LZTO/G, the two electrodes were extracted from the cells after cycling for 200 cycles at 1 A g^−1^. The surface of the LZTO electrode was severely damaged with clear cracks (Fig. S9a†), which could worsen its cyclic performance. However, the active material layer stayed integrated for the LZTO/G electrode (Fig. S9b†), which can form a good conductive layer. Moreover, compared with that observed for the LZTO electrode, there was better adhesion between the LZTO/G active material coating and the Cu current collector (Fig. S9c and d†), which could help maintain a whole conductive network.

## Conclusions

An LZTO/G nanocomposite has been successfully synthesized by a two-step reaction. LZTO particles can be well dispersed into the G conductive network. The structure is beneficial for the insertion and deinsertion of Li^+^ ions. G having outstanding electronic behavior can improve the electronic conductivity of LZTO/G, which exhibits large specific capacities and good cycling performance at high current densities. This method is simple as well as efficient and can be extended to the preparation of other electrode materials.

## Conflicts of interest

There are no conflicts to declare.

## Supplementary Material

RA-008-C8RA05893H-s001
