# Supplementary material for: Li2ZnTi3O8/graphene nanocomposite as a high-performance anode material for lithium-ion batteries
Source: RSC Adv. 2018 Sep 10;8(55):31628–32. doi: 10.1039/c8ra05893h (PMC9085565; doi:10.1039/c8ra05893h)
Supplement: RA-008-C8RA05893H-s001 [file RA-008-C8RA05893H-s001.pdf]

Electronic Supplementary Material (ESI) for RSC Advances.
This journal is © The Royal Society of Chemistry 2018

$Li_2ZnTi_3O_8$/graphene nanocomposite as a high performance anode material for lithium ion batteries

Song Wang,[a] Lijuan Wang,[a,b,]* Zhaohui Meng,[b,]** and Baomin Luo[b]

[a] School of Chemistry and Material Science, Liaoning Shihua University, Fushun 113001, Liaoning, China

[b] College of Chemistry and Pharmaceutical Engineering, Nanyang Normal University, Nanyang 473061, Henan, China

*Corresponding author:

Lijuan Wang

[a] School of Chemistry and Material Science, Liaoning Shihua University, Fushun 113001, Liaoning, China

[b] College of Chemistry and Pharmaceutical Engineering, Nanyang Normal University, Nanyang 473061, Henan, China

E-mail address: lijuanw123@163.com

Tel: +8624-56861711; Fax: +8624-56861709

Experimental

Synthesis of graphene oxide (GO) sheets

Graphite powder (6 g) was added into the solution of concentrated H_2SO_4 (24 mL), $K_2S_2O_8$ (5 g), and P_2O_5 (5 g) at 80 ℃. The mixture was kept at 80 ℃ for 4.5 h, cooled to room temperature, diluted with deionized water (DI, 1 L), left overnight, filtered and then dried at 60 ℃ in vacuum.

The preoxidized graphite was added into concentrated H_2SO_4 (240 mL) in an ice bath, and then $KMnO_4$ (30 g) was slowly added in the mixture above under stirring to promote the further oxidation of graphite with the temperature below 20 ℃. Then, the mixture was stirred for 2 h in an ice bath to keep the temperature at 35 ℃, diluted with DI water (500 mL) in the same bath to keep the temperature below 50 ℃, stirred for another 2 h, and further diluted with DI water (1.4 L). 30% H_2O_2 (40 mL) was then immediately added into the mixture to form a brilliant yellow product which was filtered and washed with 1:10 HCl (2 L) aqueous solution and DI water (2 L). The resulting GO sheets were ultrasonically dispersed for 0.5 h, filtered and dried at 60 ℃ in vacuum.

The dispersed GO sheets (2 g) were ball-milled for 38 h using ethanol (20 mL) as the dispersing medium with 400 r min^{-1} and then dried at 60 ℃ in vacuum to form the final GO sheets. The mass ratio of the mixture to balls is 1:20. The vessel of the ball-milling jar is 100 mL.

Physical and electrochemical performance measurements

Thermogravimetric (TG) and carbon content analyses were conducted on a RD496 thermal analyzer from room temperature to 900 °C and to 800 °C in air,

respectively. The phases were investigated via a X-ray diffraction technique conducted on a Bruker D8 Advance X-ray diffractometer with Cu Kα radiation (λ = 1.54 Å) in the 2θ ranges of 5-85 ° for LZTO/G as well as LZTO, and 5-40 ° for GO as well as G with 4 ° min^{-1}. The morphologies of the products and the electrodes were observed by SU8220 and S4800 scanning electron microscopes (SEM), respectively. The high-resolution transmission electron microscope (HR-TEM) (FEI Tecnai F20) was used to observe the nanoscale microstructures. The specific surface areas and pore size distributions were measured via N_2 adsorption on a specific surface area and pore size distribution analyzer (3H-2000PS2). A four-probe system (SB100A/2) was used to measure the electronic conductivity. Fourier transform infrared (FTIR) spectra were obtained from a TENSOR27 FTIR spectrometer. X-ray photoelectron spectroscopy (XPS) measurements (PHI 5600 CI, mono-chromatic Al-Ka radiation) were used to identify the surface species. Raman spectra were recorded on a micro-Raman spectroscope (Renishaw 1000) with an excitation wavelength of 532 nm.

The electrochemical measurements were performed in CR2025 coin-type cells. The working electrode was composed of 85 wt.% active material for LZTO or LZTO/G, 10 wt.% conductive agent of acetylene black, and 5 wt.% binder of polyvinylidene difluoride (PVDF). Charge-discharge and cyclic voltammetry (CV) measurements were in the range of 0.02-3.0 V. Electrochemical impedance spectroscopies (EIS) were recorded with an *ac* voltage of 5 mV from 10 mHz to 100 kHz.

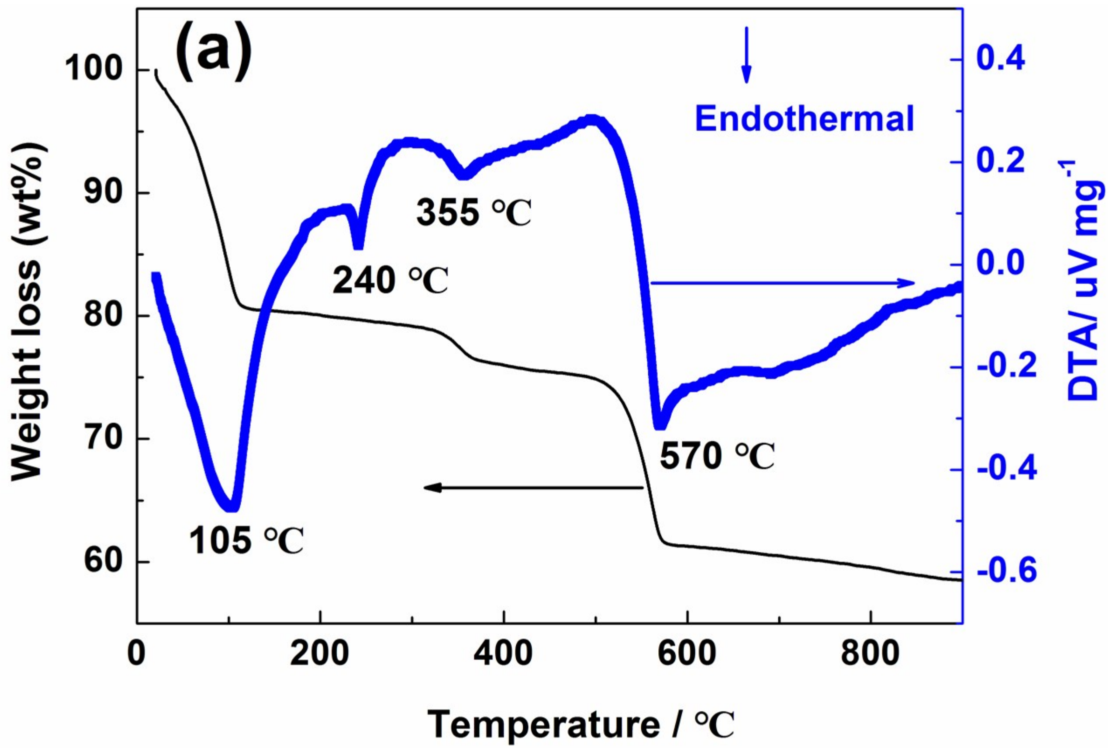
(a)
Endothermal
355 °C
240 °C
105 °C
570 °C
Weight loss (wt%)
DTA/ uV mg-1
Temperature / °C
100
90
80
70
60
0
200
400
600
800
0.4
0.2
0.0
-0.2
-0.4
-0.6

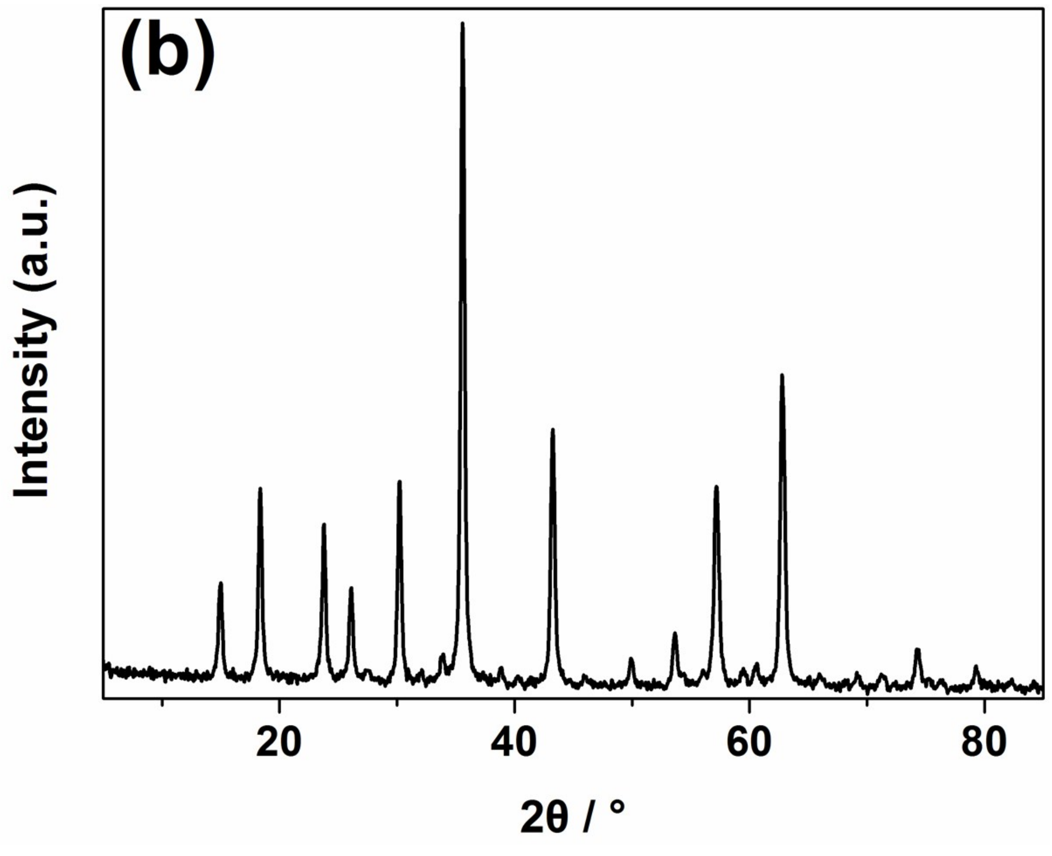
(b)
Intensity (a.u.)
2θ / °
20
40
60
80

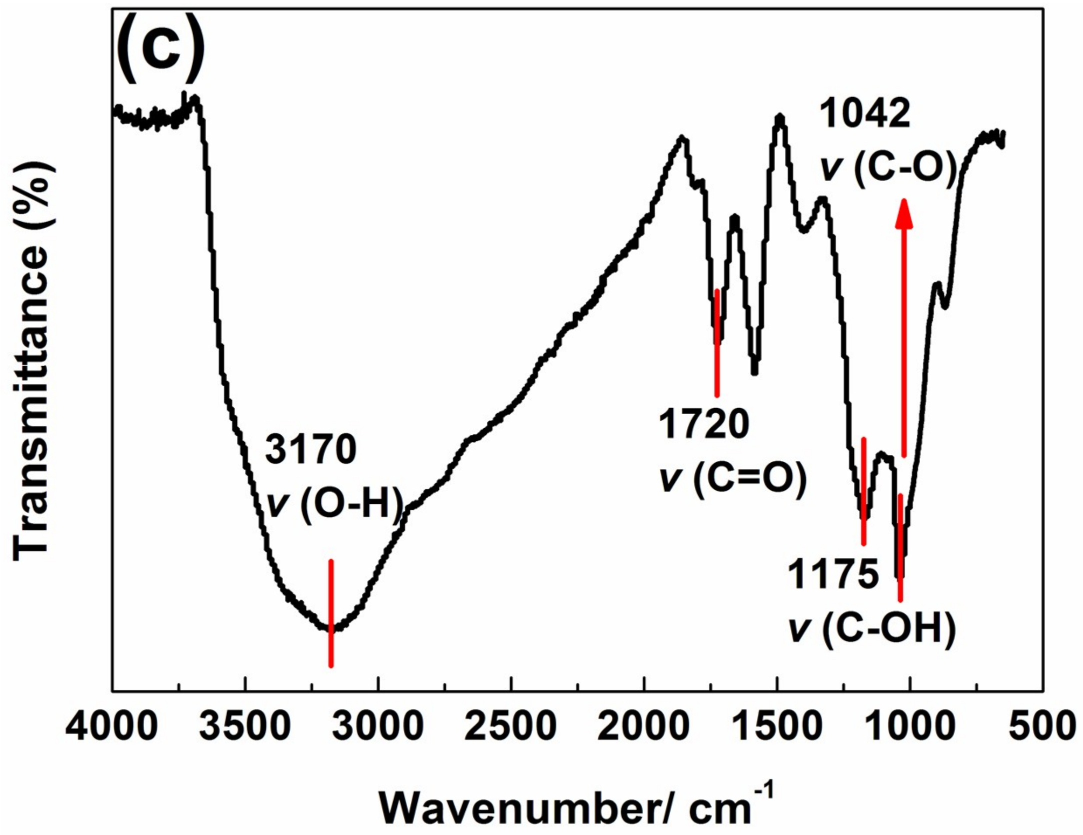

Fig. S1 (a) TG-DTA curves of the precursor of $Li_2ZnTi_3O_8$; (b) XRD pattern of the precursor for $LiOH{\cdot}H_2O$, $LiNO_3$, ZnO and TiO_2 preheated at 250 °C for 3 h and then 600 °C for 4 h in air; (c) FT-IR spectrum of GO.

Fig. S1a shows the TG-DTA curves of the precursor for $Li_2ZnTi_3O_8$ at a heating rate of 10 °C min^{-1} from room temperature to 900 °C in air. It can be seen that there are three obvious weight losses during heating the precursor, corresponding to the three peaks on the DTA curve. The weight loss in the range from room temperature to 100 °C may be related to the evaporation of absorbed water from the precursor. Subsequently, the weight loss from 320 to 370 °C may originate from the loss of crystal water from $LiOH{\cdot}H_2O$. In the range of 500-570 °C, a sharp weight loss associated with the decomposition of LiOH and $LiNO_3$ appears on the TG curve. When the temperature exceeds 570 °C, a platform appears on the TG curve, indicating

zero weight loss and the formation of a relatively stable material after 570 °C checked by XRD shown in Fig. S1b.

Fig. S1c shows the Fourier transform infrared (FT-IR) spectrum of GO. The observed representative peaks of GO include the bands at 1042 cm^{-1} (C-O stretching vibration of epoxide), 1175 cm^{-1} (C-OH stretching for phenolic), and 1720 cm^{-1} (C=O stretching of carbonyl and carboxyl groups located at the edges of the GO networks). These characteristic peaks of GO confirm the presence of the oxygen-containing functional groups in carbon frameworks.

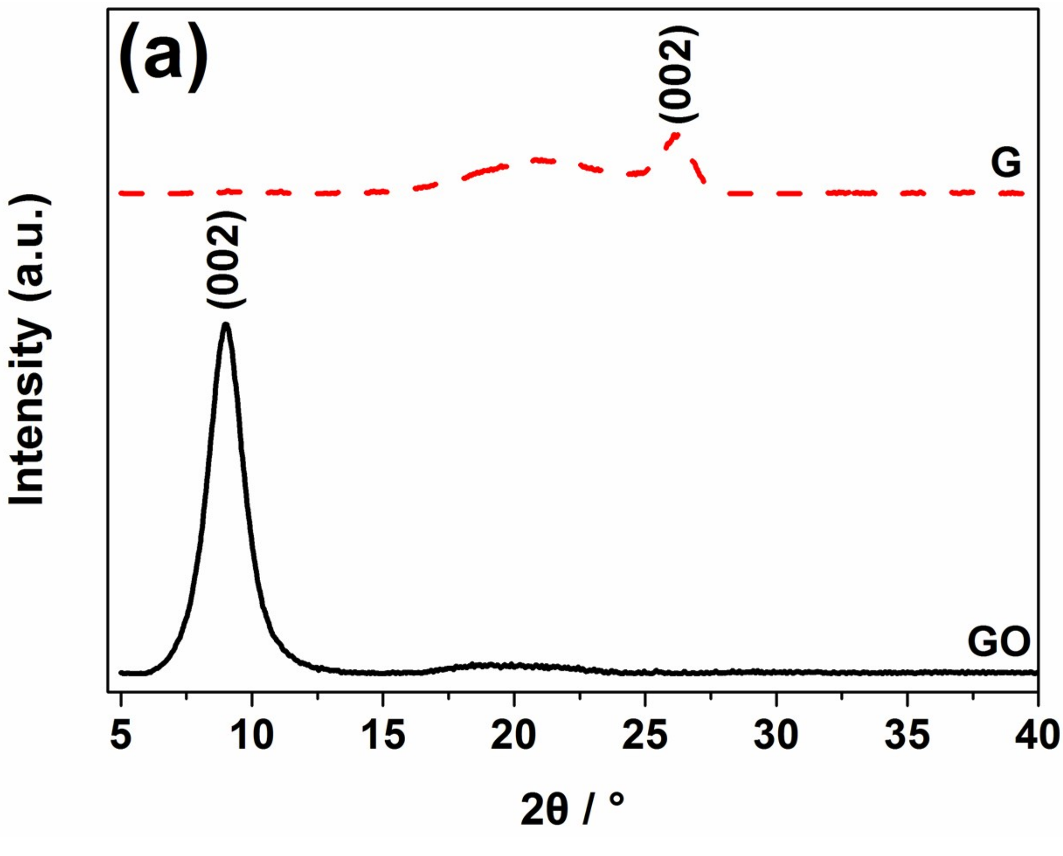

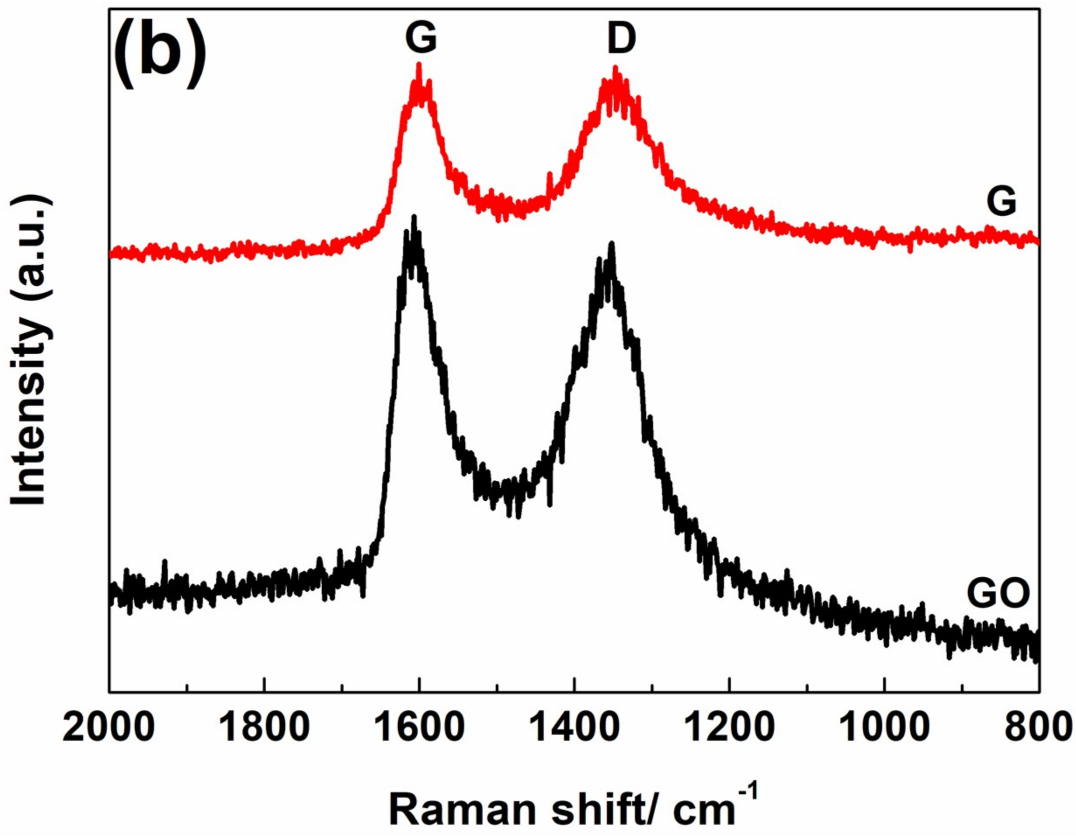
(b)
G
D
G
GO
Intensity (a.u.)
2000
1800
1600
1400
1200
1000
800
Raman shift/ cm-1

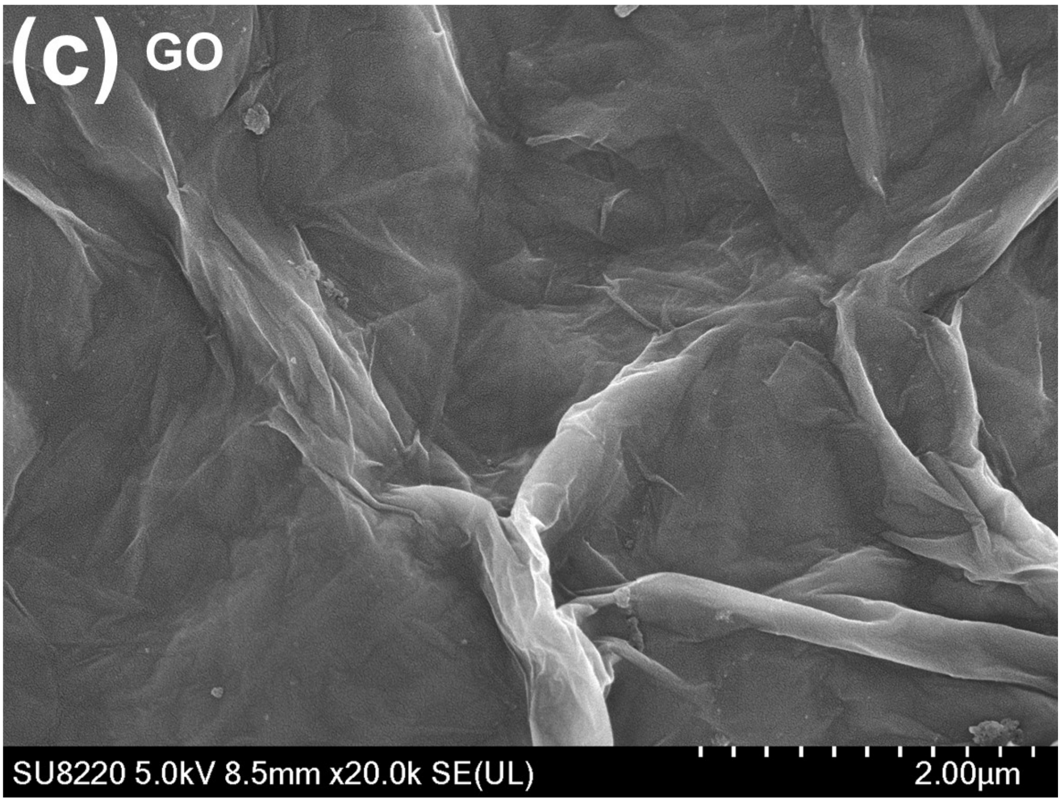
(c) GO
SU8220 5.0kV 8.5mm x20.0k SE(UL)
2.00μm

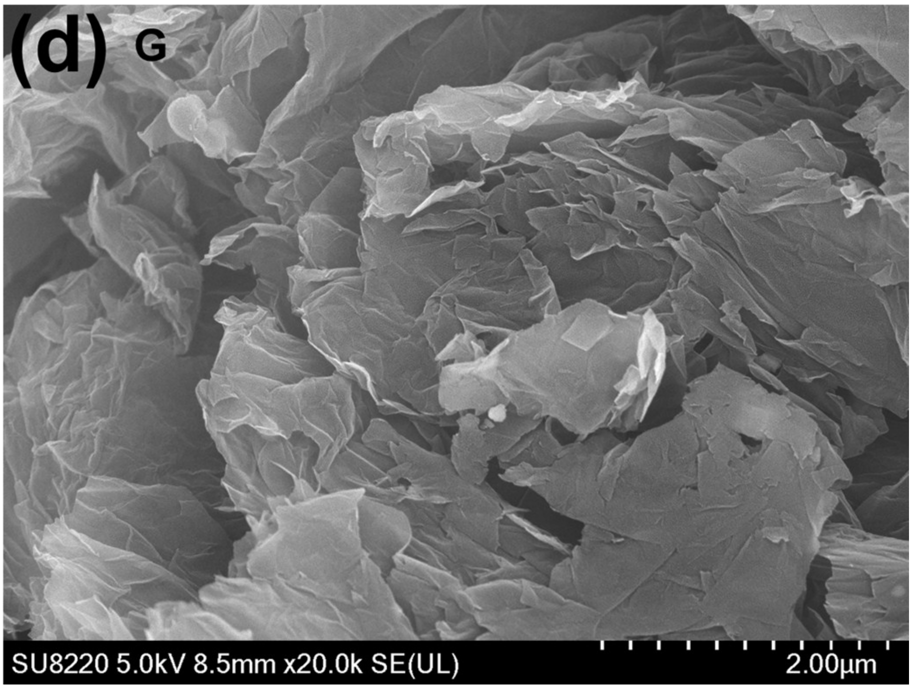
(d) G
SU8220 5.0kV 8.5mm x20.0k SE(UL)
2.00μm

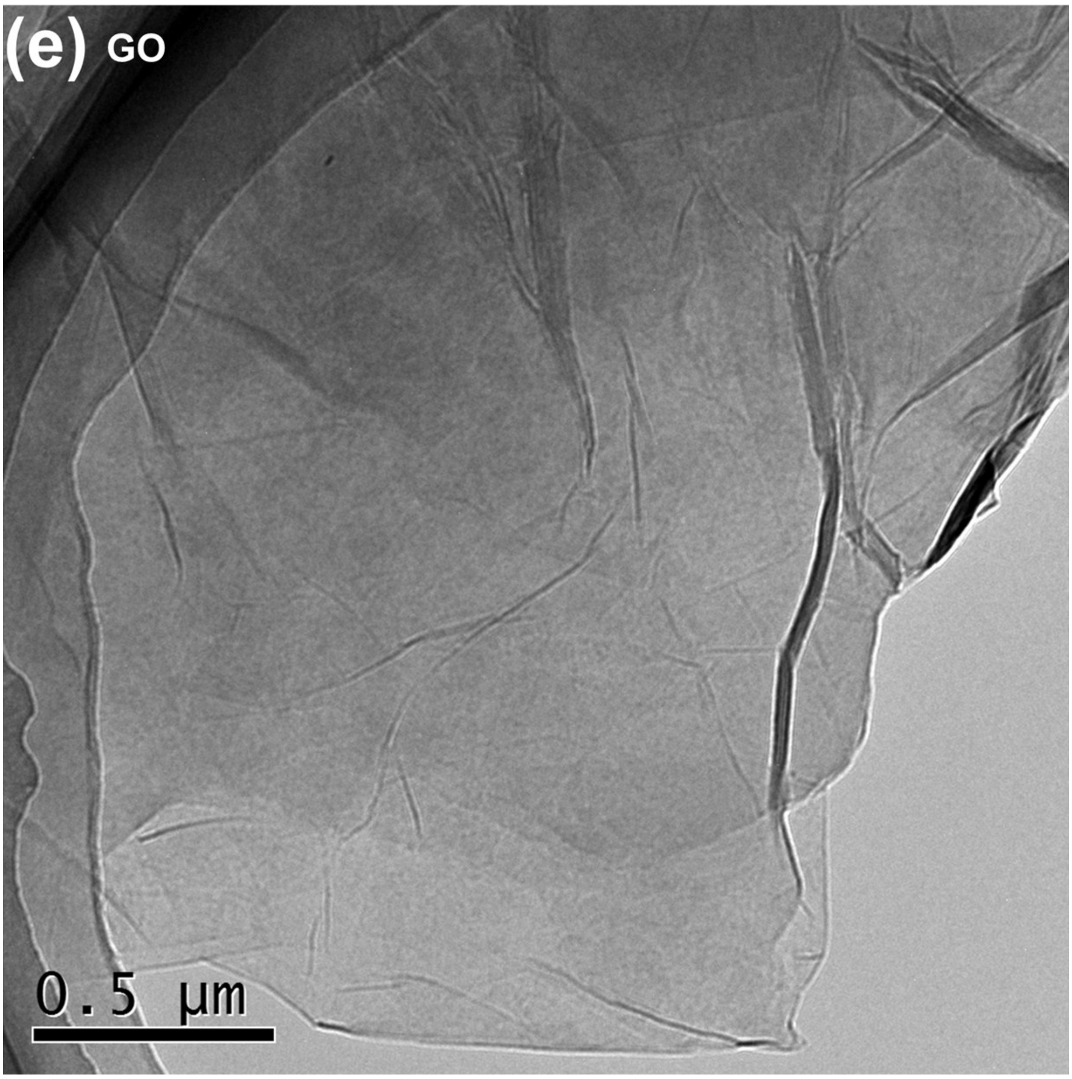
(e) GO
0.5 μm

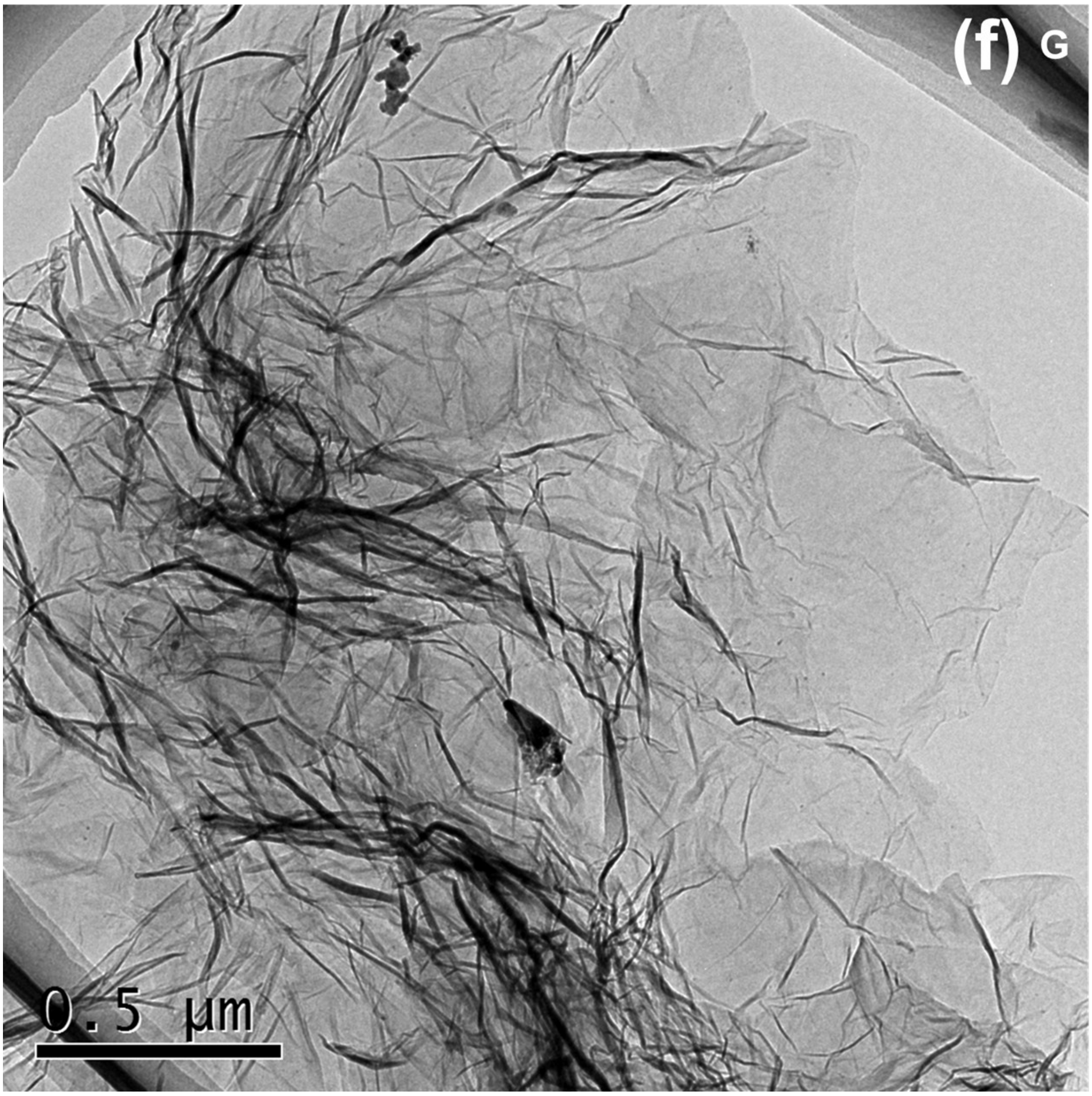

Fig. S2 (a) X-ray diffraction patterns, (b) Raman spectra, (c-d) SEM images and (e-f) TEM images of GO and G.

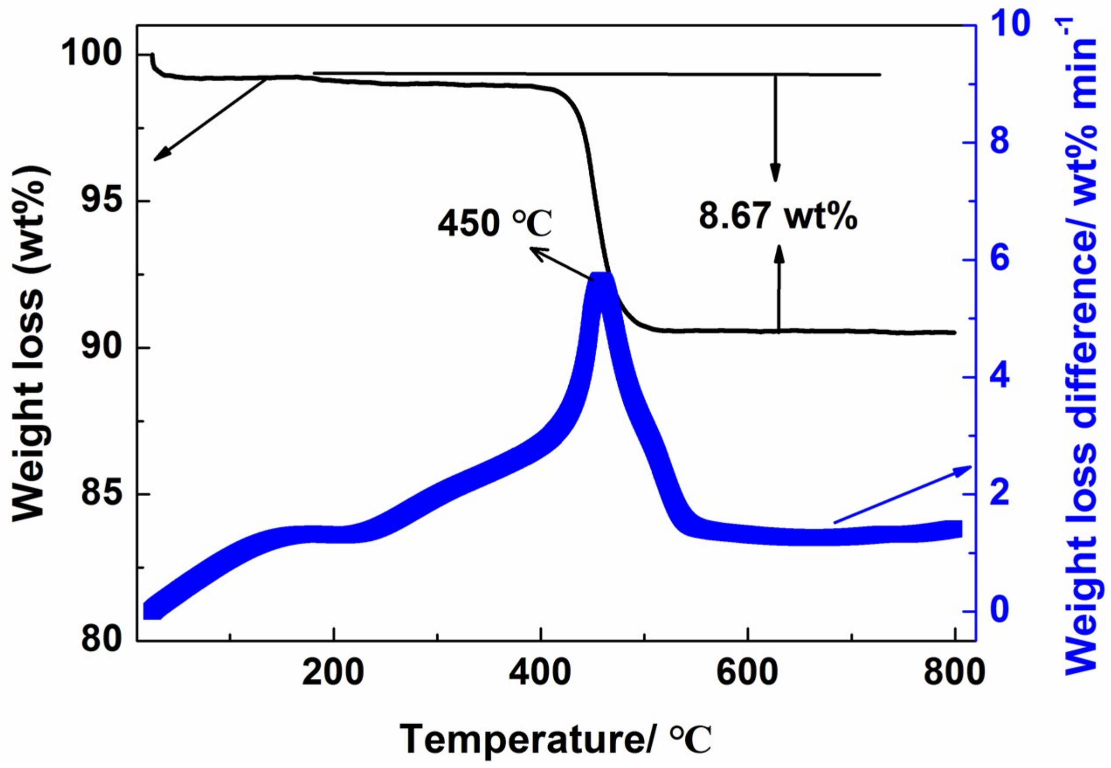

Fig. S3 TG-DTG curves of LZTO/G composite.

The G content in LZTO/G composite quantified by TG measurement is shown in Fig. S3. The evaporation of adsorbed water from the composite occurs from room temperature to 150 °C with the slow weight loss of 1.0-2.0 wt.%. The oxidation of carbon in air occurs from 300 to 510 °C with sharp weight loss. No weight loss occurs above 510 °C, indicating that the parent LZTO is stable. According to the TG result, the G content is 8.67 wt%.

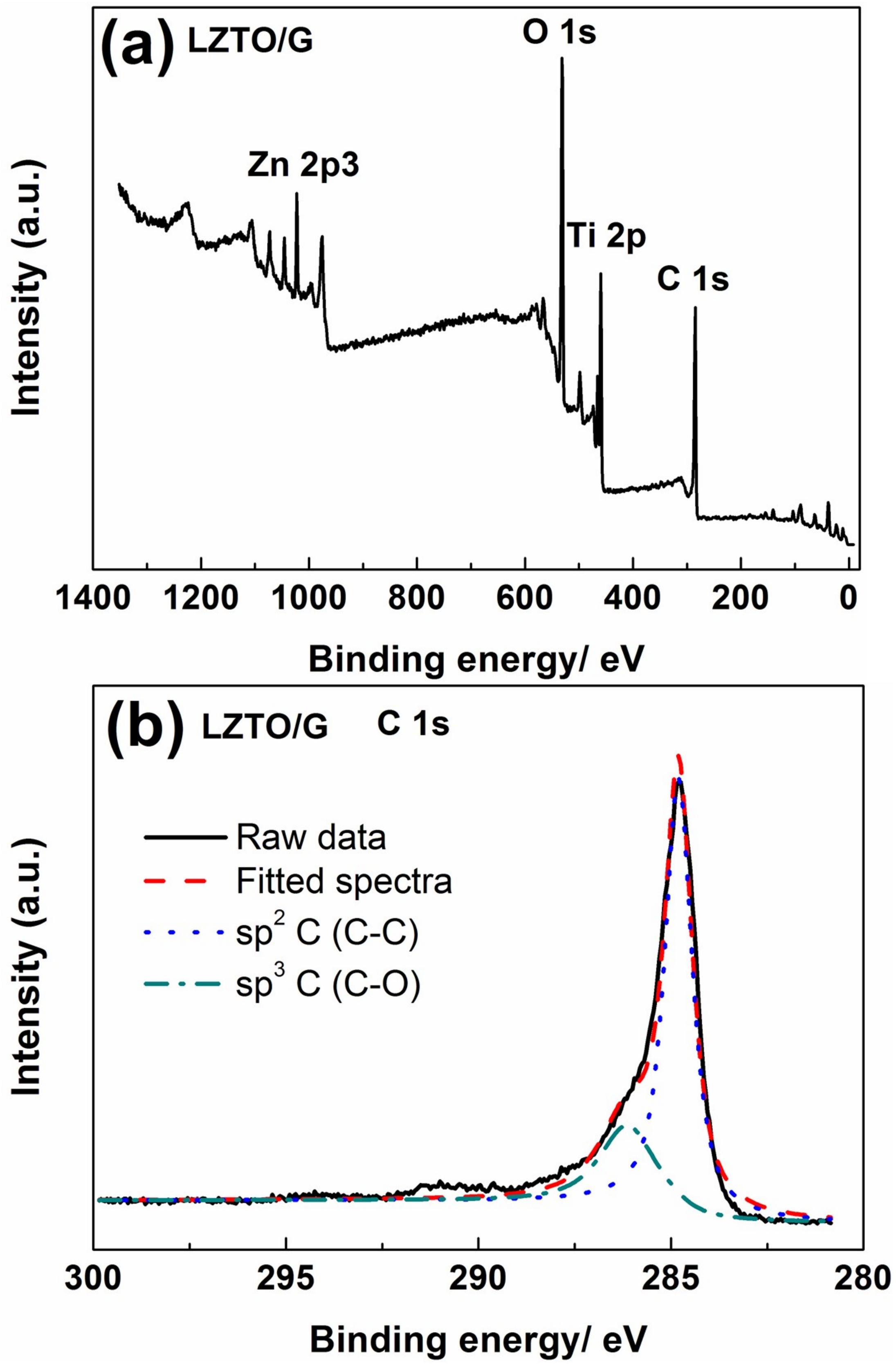
(a)
LZTO/G
O 1s
Zn 2p3
Ti 2p
C 1s
Intensity (a.u.)
1400
1200
1000
800
600
400
200
0
Binding energy/ eV
(b)
LZTO/G
C 1s
Raw data
Fitted spectra
sp2 C (C-C)
sp3 C (C-O)
Intensity (a.u.)
300
295
290
285
280
Binding energy/ eV

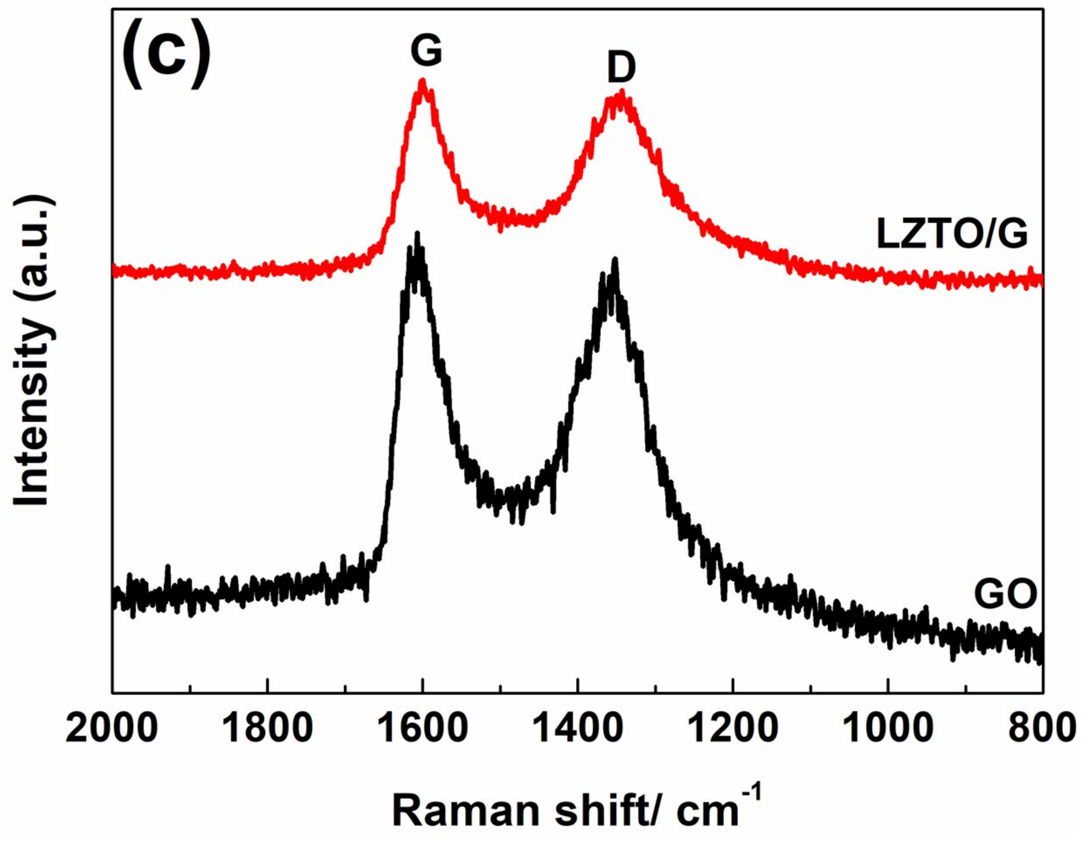

Fig. S4 (a) XPS spectrum and (b) high resolution C 1s XPS spectrum of LZTO/G; (c) Raman spectra of LZTO/G and GO.

The XPS spectrum (Fig. S4a) confirms the existence of Zn, Ti, O and C elements in LZTO/G. As shown in Fig. S4b, the high-resolution XPS spectrum of C 1s can be fitted into two peaks at 284.8 and 286.1 eV corresponding to C-C and C-O, respectively. The existence of C-C with high amount demonstrates that GO was reduced during the calcination. In addition, the I_D/I_G value of LZTO/G is larger than that of GO obtained from Raman spectra (Fig. S4c), which further confirms that GO was reduced.

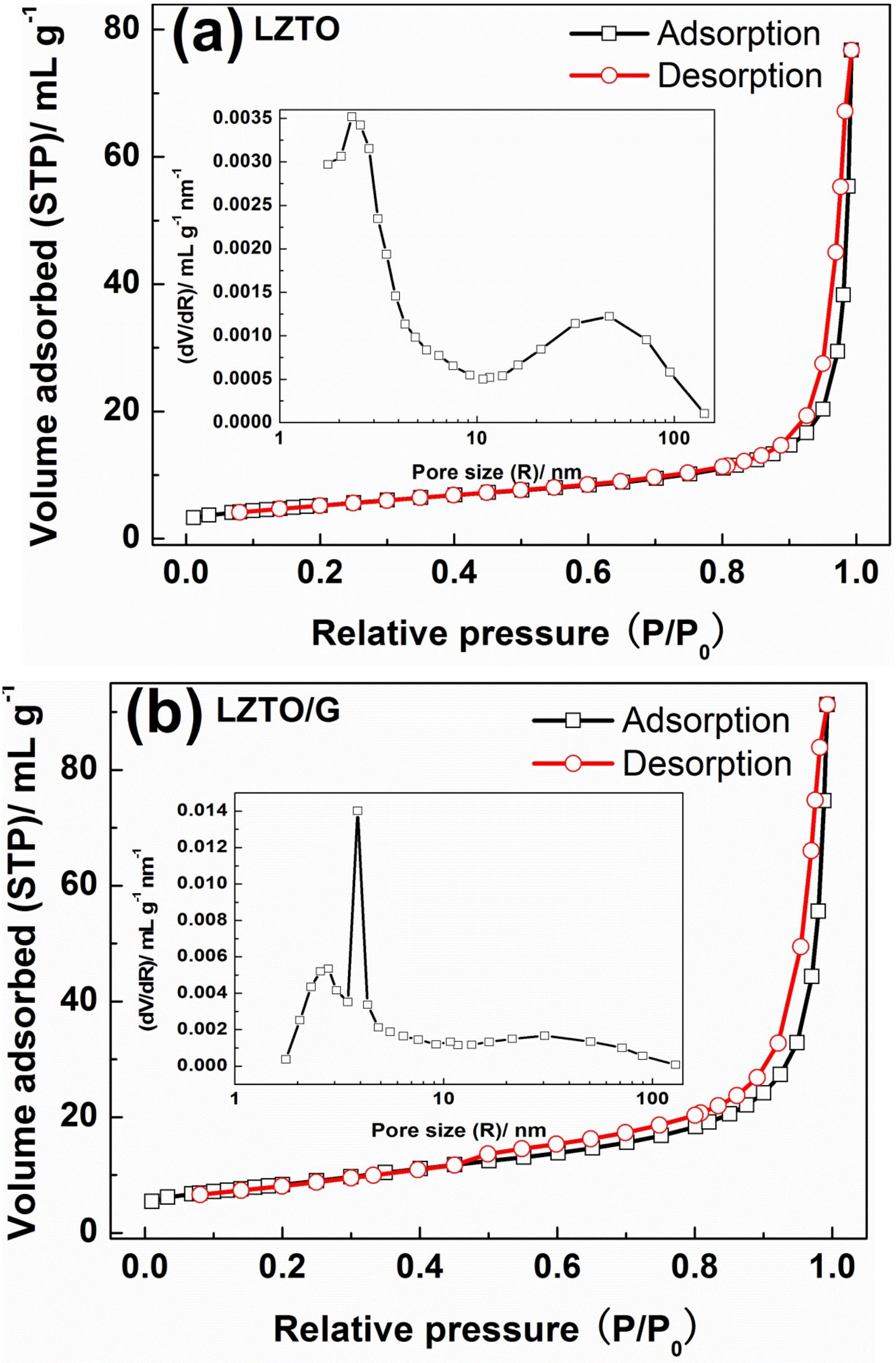

Fig. S5 N_2 adsorption-desorption isotherms of (a) LZTO and (b) LZTO/G (Insets, the pore size distributions of the LZTO and LZTO/G anode materials).

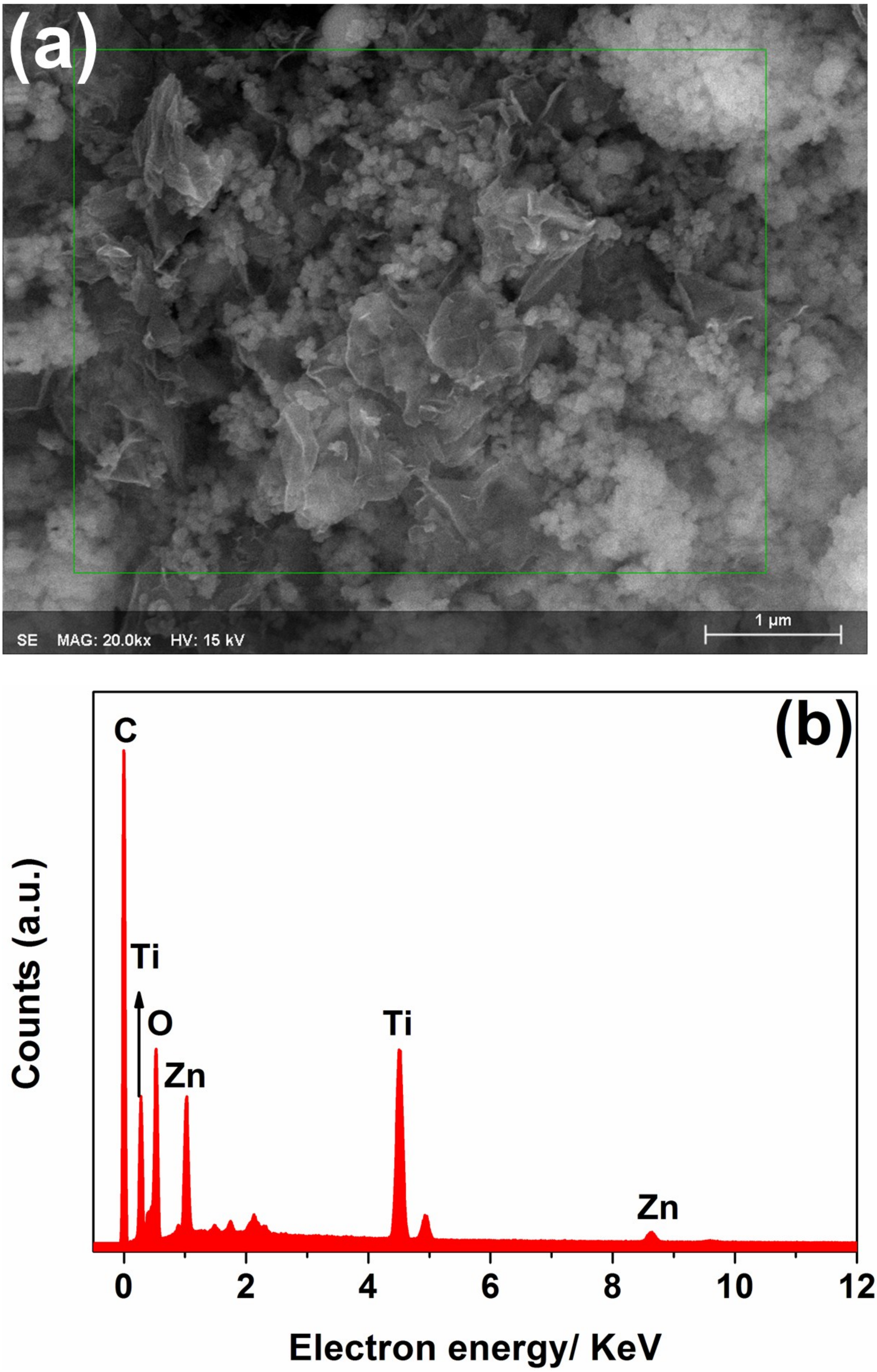
(a)
SE MAG: 20.0kx HV: 15 kV
1 μm
(b)
C
Ti
O
Zn
Ti
Zn
Counts (a.u.)
0
2
4
6
8
10
12
Electron energy/ KeV

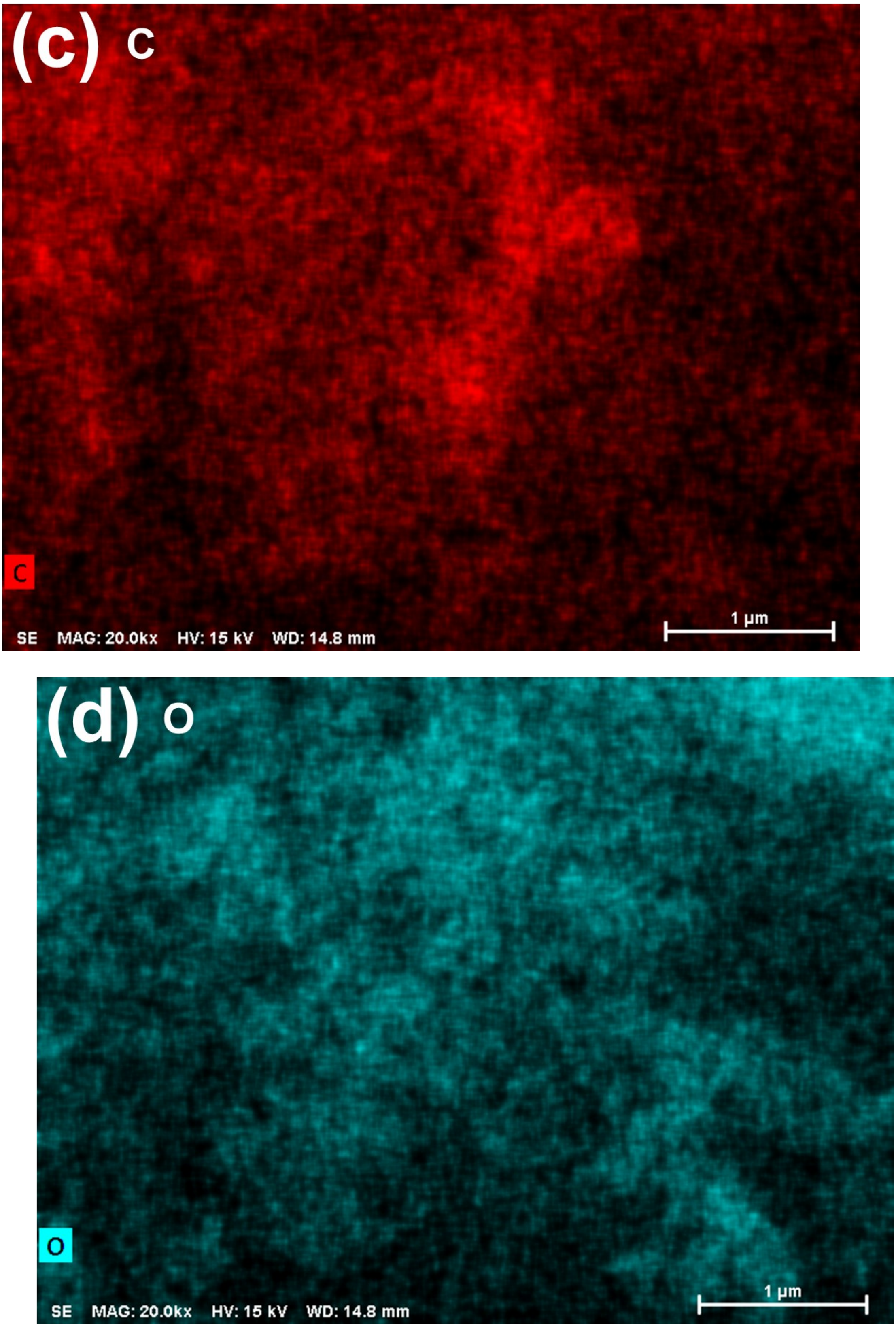
(c) C
C
SE MAG: 20.0kx HV: 15 kV WD: 14.8 mm
1 µm
(d) O
O
SE MAG: 20.0kx HV: 15 kV WD: 14.8 mm
1 µm

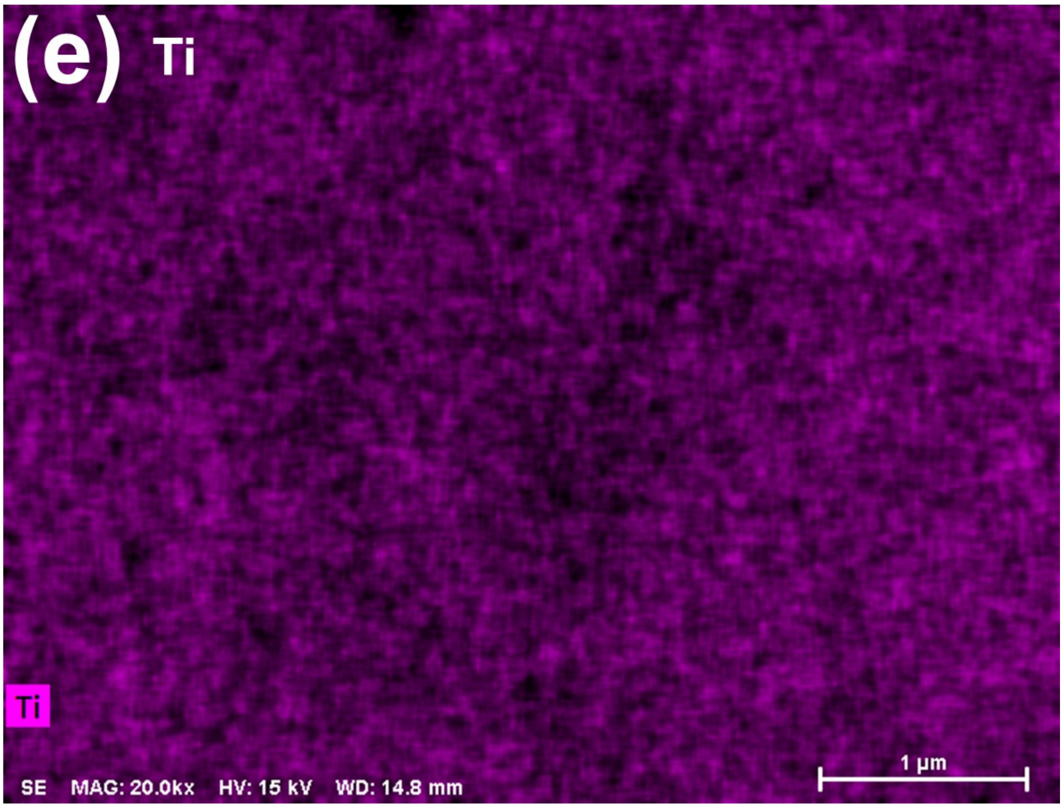

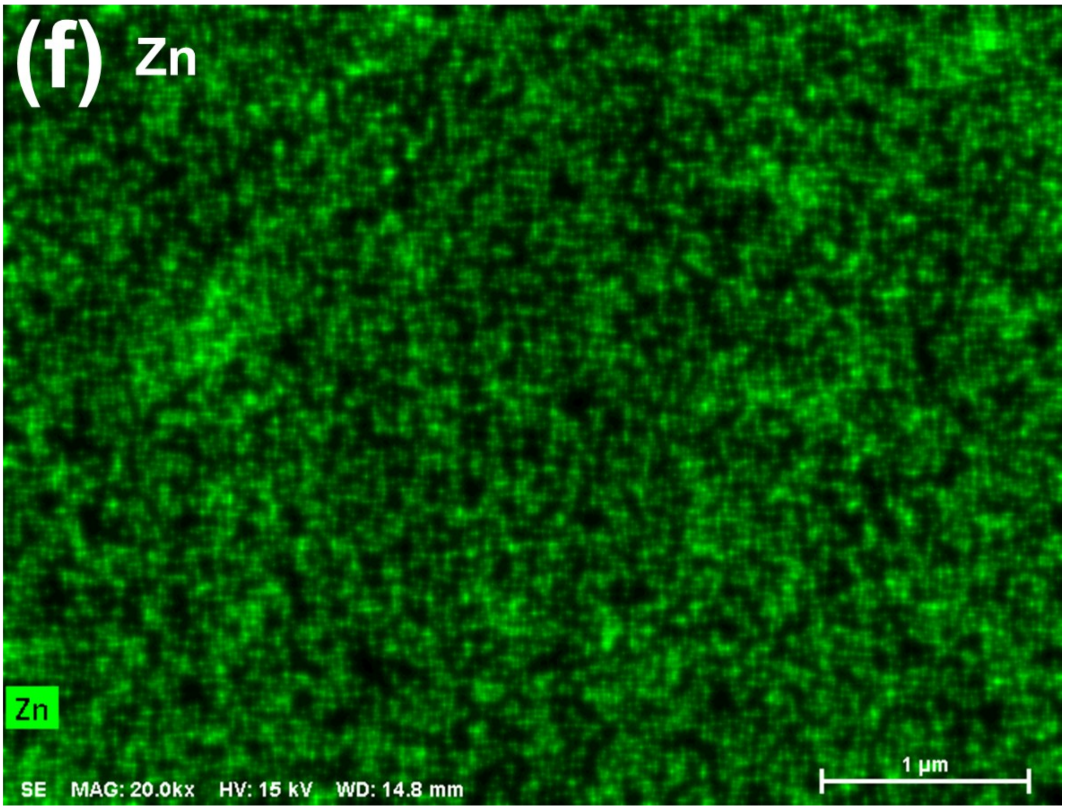

Fig. S6 (a) SEM image and (b) EDS spectrum of LZTO/G particles. Corresponding EDS element mappings of (c) C, (d) O, (e) Ti and (f) Zn in the LZTO/G particles.

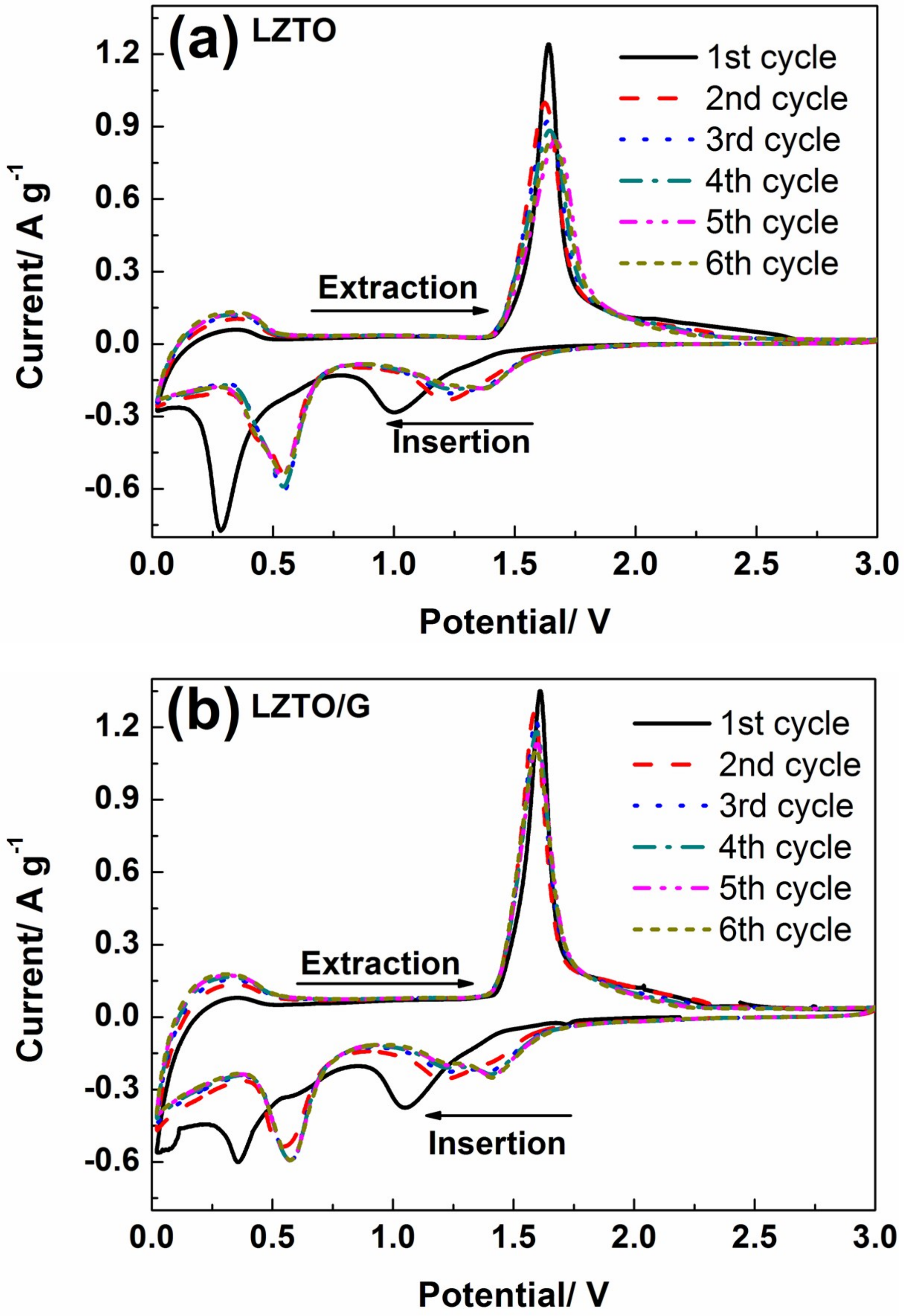
(a) LZTO
1st cycle
2nd cycle
3rd cycle
4th cycle
5th cycle
6th cycle
Extraction
Insertion
Current/ A g-1
Potential/ V
1.2
0.9
0.6
0.3
0.0
-0.3
-0.6
0.0
0.5
1.0
1.5
2.0
2.5
3.0
(b) LZTO/G
1st cycle
2nd cycle
3rd cycle
4th cycle
5th cycle
6th cycle
Extraction
Insertion
Current/ A g-1
Potential/ V

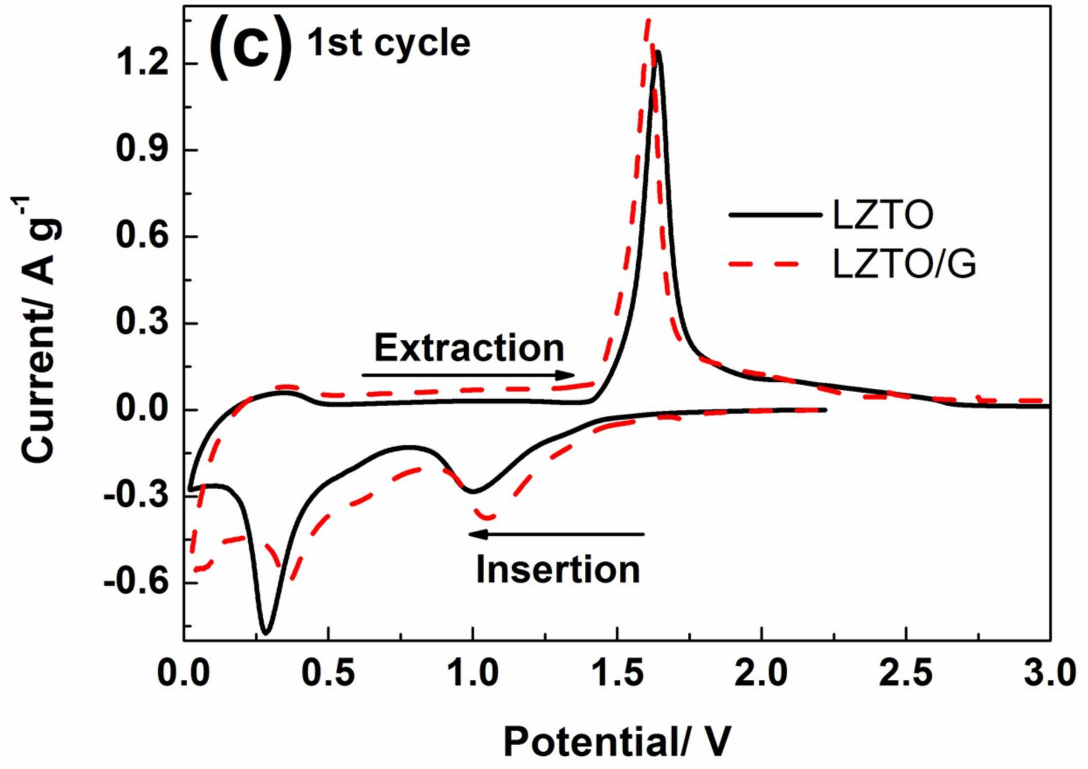

Fig. S7 Cyclic voltammograms of (a) LZTO and (b) LZTO/G electrodes from the 1st to the 6th cycle, and (c) comparison of cyclic voltammograms for LZTO and LZTO/G electrodes for the 1st cycle at a rate of 0.5 mV s^{-1} in the range of 0.02-3.0 V (vs. Li/Li^+).

The CV curves of the LZTO and LZTO/G were recorded at 0.5 mV s^{-1} in the potential range of 0.02-3.0 V and are shown in Fig. S7. There is a pair of cathodic and anodic peaks in the range of 1.0-2.0 V for each sample, corresponding to the Ti^{4+}/Ti^{3+} redox couple. The cathodic peak below 0.5 V originates from multiple restoration of Ti^{4+} as previous reports [S1,S2]. It is noted that, for each electrode, the reduction process differs between the initial and subsequent cycles, originating from the activation and/or polarization of the electrode [S3,S4]. The potentials of the anodic peak and the cathodic peak (φ_{pa} and φ_{pc}), and the difference between anodic and cathodic peak potentials (φ_p) are listed in Table S4 for the two electrodes at the 1st

cycle. Compared with LZTO electrode, LZTO/G electrode has smaller φ_p (0.553 V), indicating that the polarization is small for the electrode and the insertion and deinsertion of Li^+ ions are easy.

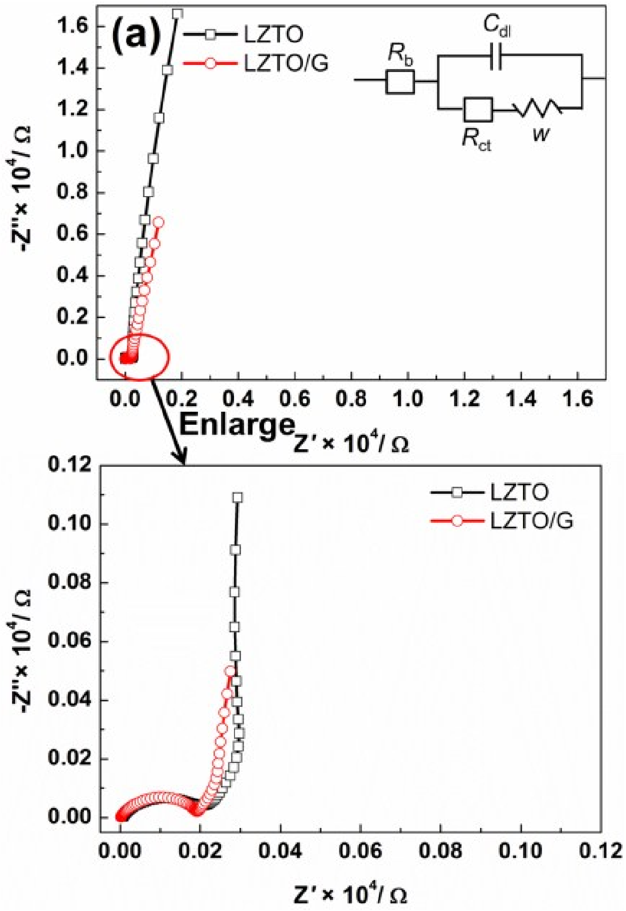

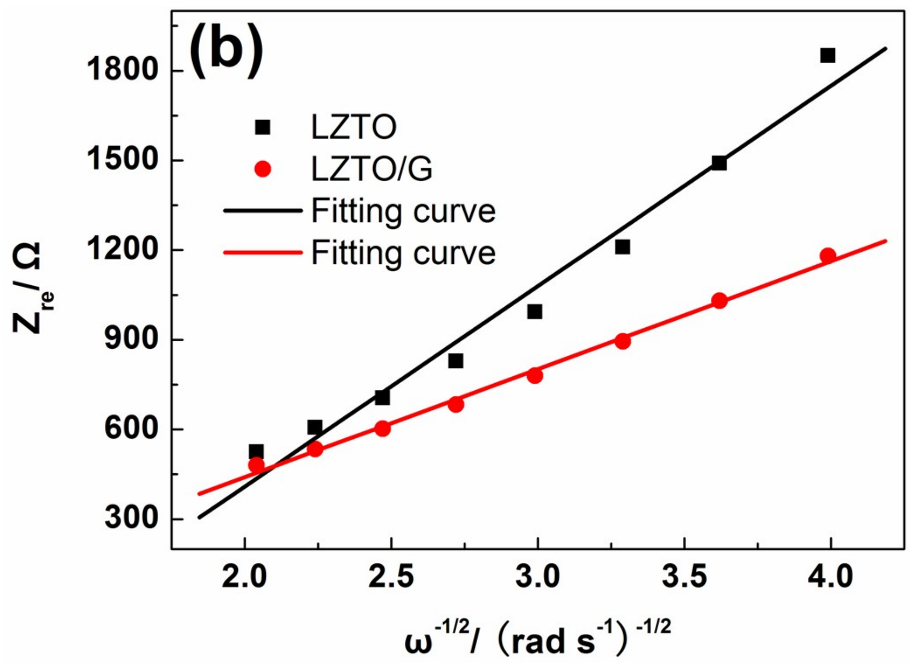

Fig. S8 (a) Impedance spectra of LZTO and LZTO/G electrodes (inset, corresponding equivalent circuit) and (b) relationship between Z_{re} and $\omega^{-1/2}$.

For the LZTO and LZTO/G electrodes, the electrochemical impedance data were collected on as assembled cells before cycling and are presented in Fig. S8a. The inset is the equivalent circuit model. R_b is the combined impedance of the electrolyte and cell components corresponding to the small intercept; C_{dl} and R_{ct}, are the double layer capacitance and charge transfer resistance corresponding to the semicircle, respectively; W represents Warburg impedance. Compared with LZTO electrode, the LZTO/G has smaller charge transfer resistance of 187.6 Ω (Table S6), which is advantageous to its electrochemical performance.

The diffusion coefficients of Li^+ ions in LZTO and LZTO/G are estimated based on the Warburg diffusion in low frequency using the following equation

$$D_{Li^+} = R^2T^2 / (2A^2n^4F^4C^2\sigma^2) \qquad (1)$$

where R is the gas constant (8.314 J mol^{-1} K^{-1}); T is the room absolute temperature (298.5 K); A is the surface area of the electrode (1.13 cm^2 in this work); n is the number of electrons transferred in the half reaction for the redox couple; F is Faraday constant (96,485 C mol^{-1}); C (8.5×10^{-3} mol cm^{-3}) is the concentration of Li^+ ion in the compound, and σ is the Warburg factor which obeys the following relationship:

$$Z_{re} = R_{\mathrm{e}} + R_{\mathrm{ct}} + \sigma\omega^{-1/2} \quad (2)$$

Fig. S8b shows the relationship between Z_{re} and $\omega^{-1/2}$. Based on the Eq. 1-2, the lithium diffusion coefficients (D_{Li^+}) of LZTO and LZTO/G can be calculated and the specific values are 1.06×10^{-15} and 3.65×10^{-15} cm^2 s^{-1}, respectively. Compared with LZTO electrode, LZTO/G has higher lithium ion diffusion coefficient, which indicates the fast diffusion of Li^+ ions and thus insures good rate capability of LZTO/G.

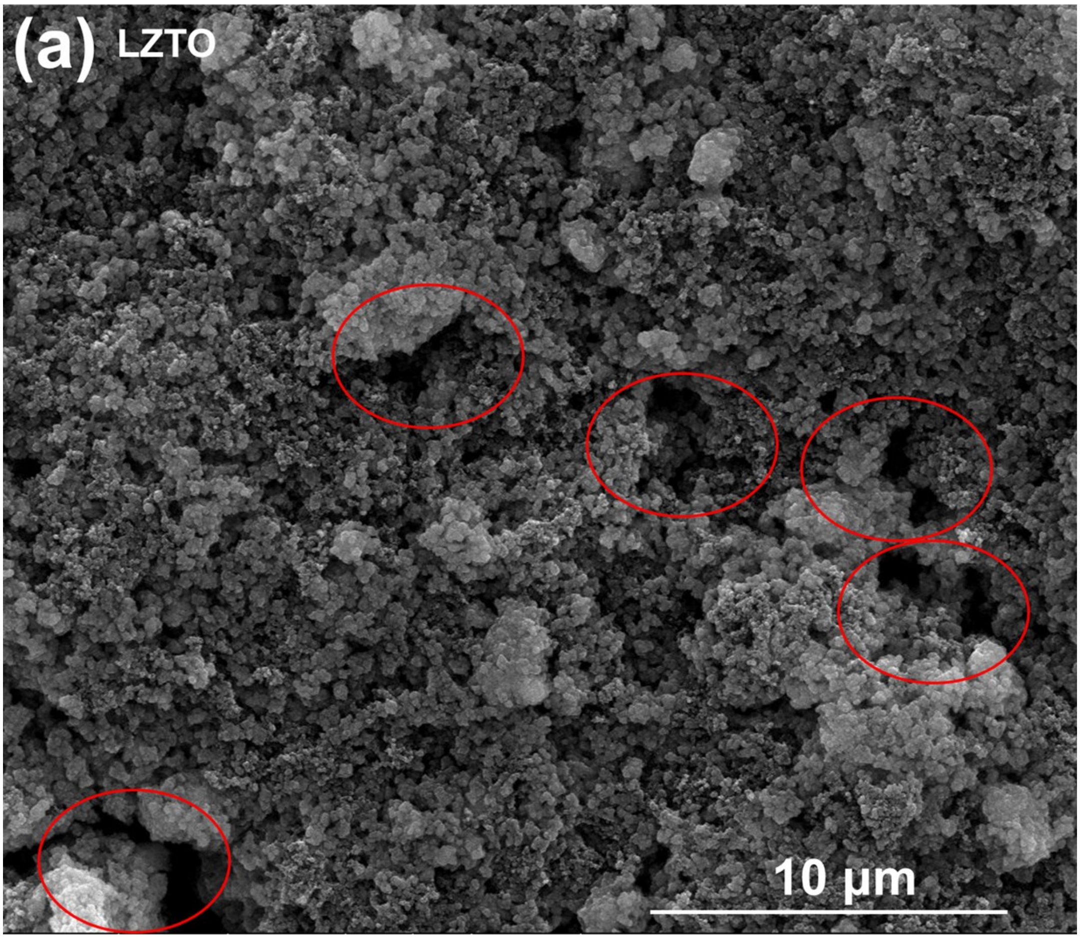
(a) LZTO
10 μm

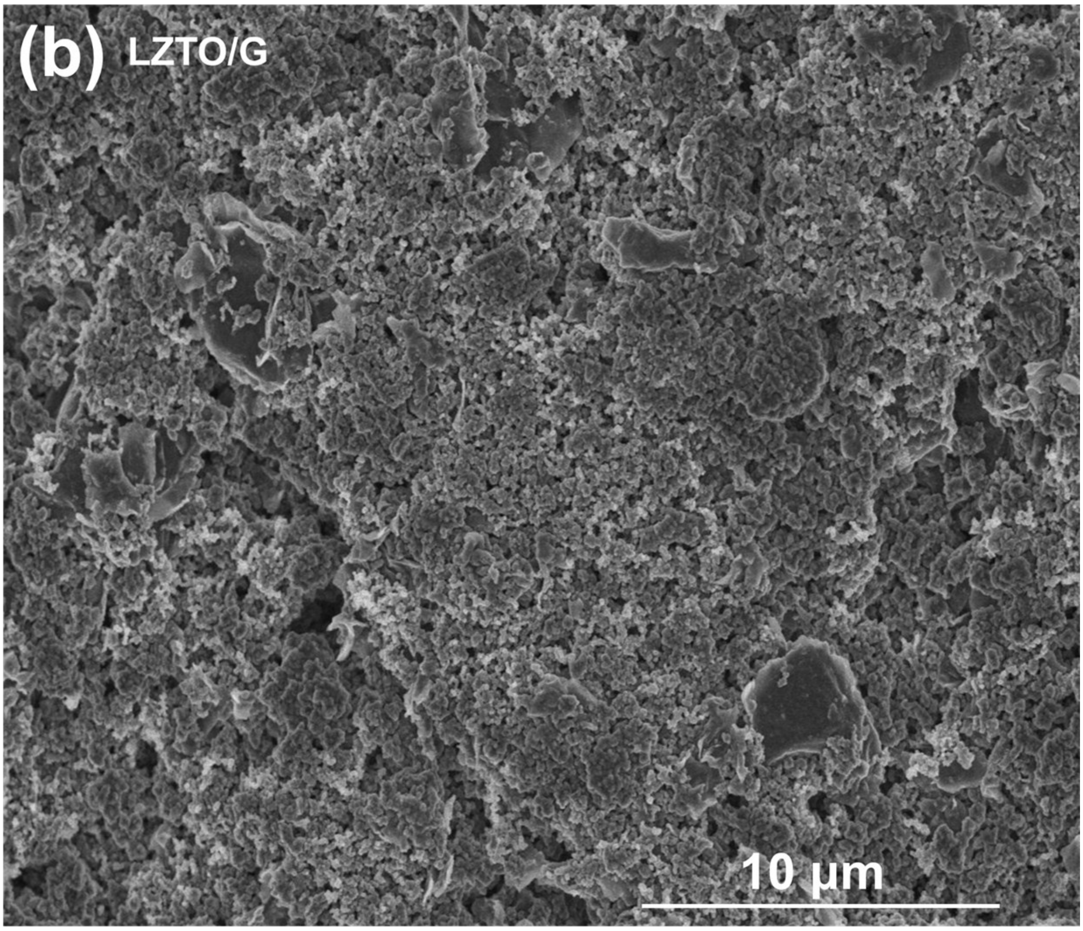
(b) LZTO/G
10 µm

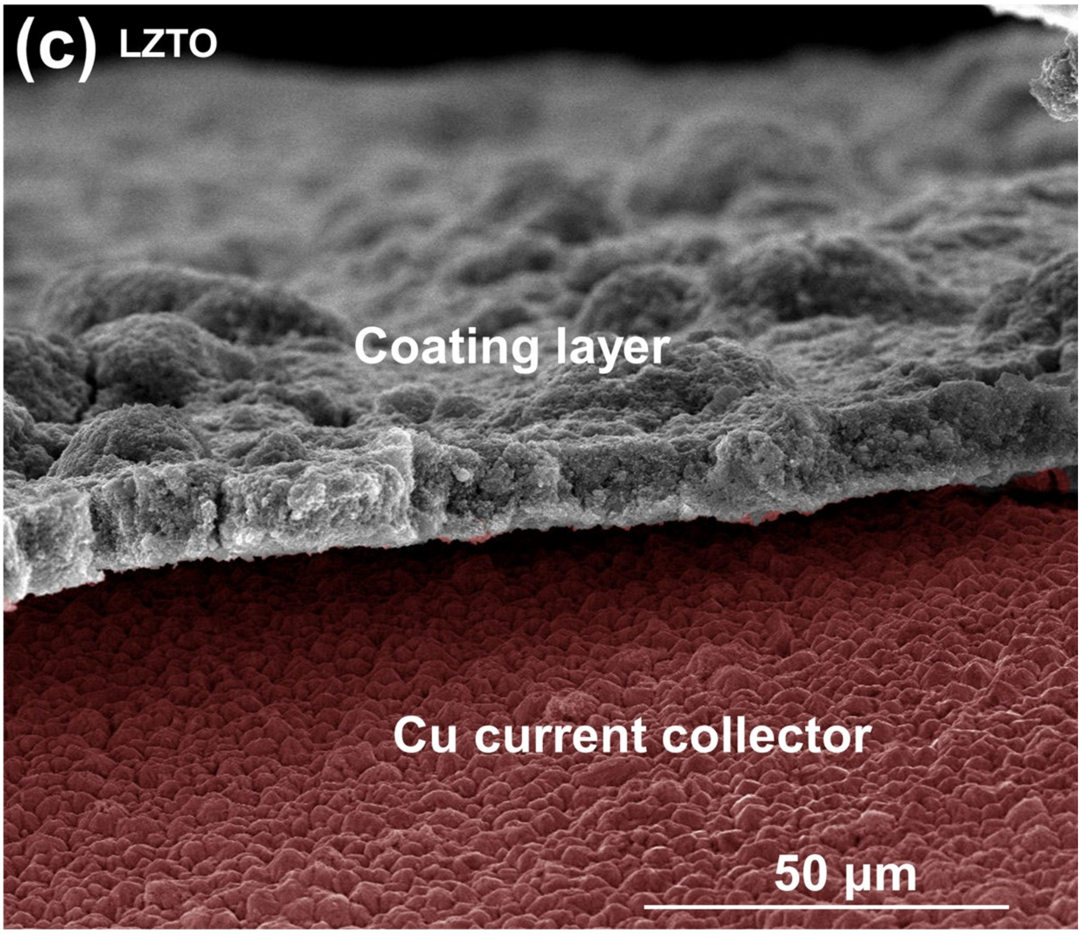
(c) LZTO
Coating layer
Cu current collector
50 µm

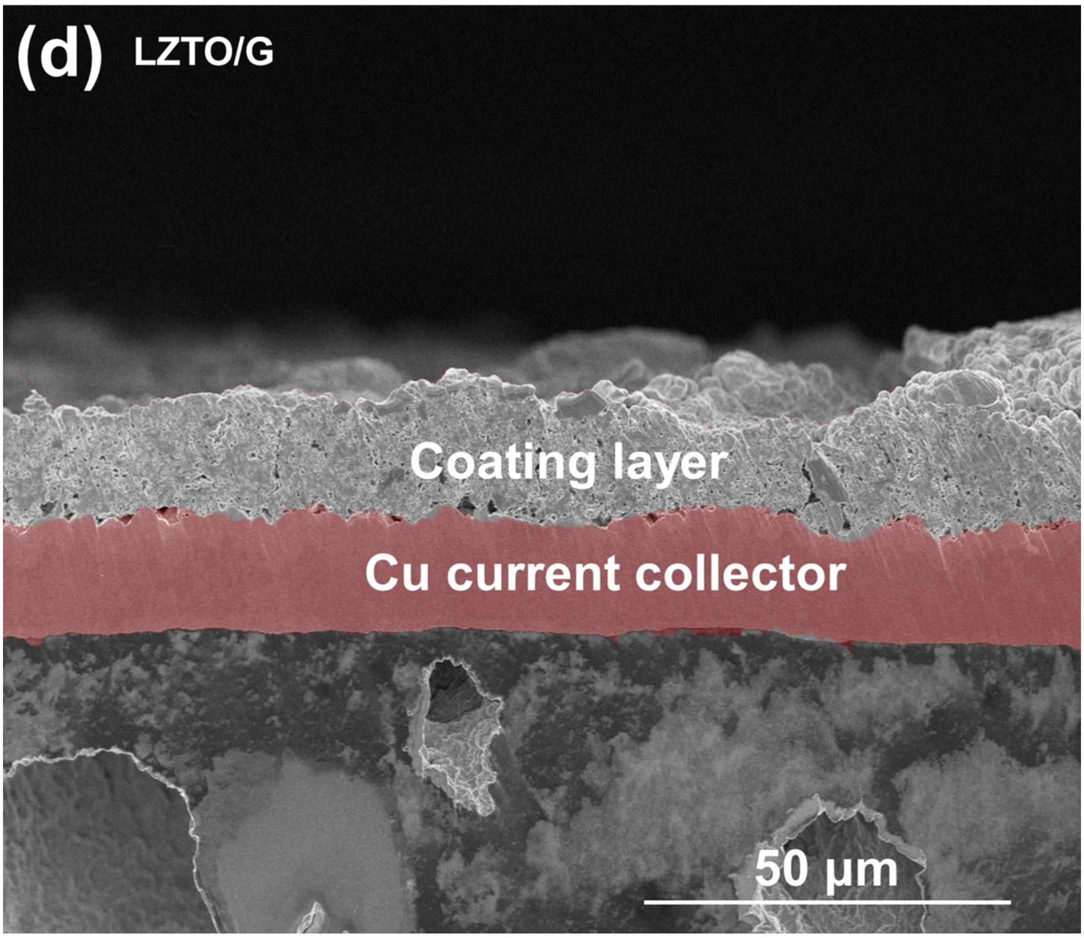

Fig. S9 SEM images of (a) LZTO and (b) LZTO/G after cycling for 200 cycles at 1 A g^{-1}; cross-sectional SEM images of (c) LZTO and (d) LZTO/G after cycling for 200 cycles at 1 A g^{-1}.

Table S1 Electronic conductivity of LZTO and LZTO/G materials.

Samples	σ (S cm^{-1})
LZTO	7.7×10^{-6}
LZTO/G	0.3767

Table S2 Lattice parameters of LZTO and LZTO/G.

Samples	*a* (Å)	*V* (Å^3)
LZTO	8.372(2)	586.8(5)
LZTO/G	8.373(9)	587.2(1)

Table S3 Specific surface areas, total pore volumes and average pore diameters of LZTO and LZTO/G.

Samples	Specific surface area (m^2 g^{-1})	Total pore volume (mL g^{-1})	Average pore diameter (nm)
LZTO	18.9	0.119	25.1
LZTO/G	30.0	0.141	18.8

Table S4 Values of the CV peaks for LZTO and LZTO/G electrodes at the first cycle.

Samples	φ_{pa} (V)	φ_{pc} (V)	φ_p (V) = φ_{pa} - φ_{pc}
LZTO	1.646	1.008	0.638
LZTO/G	1.606	1.053	0.553

Table S5 Electrochemical performance of LZTO in recent publications.

Material	Current density (A g^{-1})	2nd specific capacity (mAh g^{-1})	Cycle numbers	Capacity retention	Reference
$Li_2ZnTi_3O_8$	0.1	235	50	59.6%	[S5]
$Li_2Zn_{0.5}Cu_{0.5}Ti_3O_8$	0.1	240	50	67.5%	[S5]
$Li_2ZnTi_3O_8$+COS	1	195	550	61.5%	[S6]
$Li_2ZnTi_3O_8$+PVDF	1	160	550	25%	[S6]

LZTO-700-3	1	180.3	200	66.9%	[S7]
LZTO@C-700-1	1	236.5	200	60.8%	[S7]
LZTO@C-700-3	1	251.9	200	71.7%	[S7]
LZTO@C-700-5	1	245.7	200	69.7%	[S7]
LZTO-700-3	2	152.2	200	72.3%	[S7]
LZTO@C-700-1	2	182.8	200	67.2%	[S7]
LZTO@C-700-3	2	208.2	200	73.1%	[S7]
LZTO@C-700-5	2	207.8	200	65.7%	[S7]
LZTO@C-N-1	1	200.6	200	71.3%	[S8]
LZTO@C-N-2	1	207.9	200	77.7%	[S8]
LZTO@C-N-3	1	208.2	200	83.0%	[S8]
$Li_2ZnTi_3O_8/LiCoO_2$	2	175	550	40%	[S9]
$Li_2ZnTi_3O_8$	2	150	550	26.7%	[S9]
$Li_2ZnTi_3O_8/La_2O_3$	2	180	550	50%	[S10]
$Li_2ZnTi_3O_8$	2	160	550	25%	[S10]
$Li_2ZnTi_3O_8$	2	175	550	71.4%	[S11]
$Li_2ZnTi_{2.95}Ce_{0.05}O_8$	2.27	176.9	500	75%	[S12]
$Li_2ZnTi_3O_8$	2.27	157.1	500	22.6%	[S12]
$Li_2ZnTi_3O_8@Li_2MoO_4$	0.5/2	210	500	64.3%	[S13]
$Li_2ZnTi_3O_8@Li_2MoO_4$	0.5/3	190	500	67.9%	[S13]
$Li_2ZnTi_3O_8/C@Cu$	1	222	550	67.6%	[S14]
$Li_2ZnTi_3O_8/C$	1	220	550	45.5%	[S14]
$Li_2ZnTi_3O_8/C@Cu$	2	200	550	52.5%	[S14]
$Li_2ZnTi_3O_8/C$	2	198	550	40.4%	[S14]
LZTO-700-3	2	163.3	100	84.4%	[S15]
LZTO-700-3	3	134.9	100	84%	[S15]
LZTO/G	1	221.4	400	76.4%	The work
LZTO/G	2	213.9	300	72.3%	The work
LZTO/G	3	207.2	300	72.7%	The work
LZTO/G	5	201.2	200	71.7%	The work
LZTO/G	6	161.5	200	83.5%	The work

Table S6 Impedance parameters calculated from equivalent circuit model and lithium ion diffusion coefficient.

Samples	R_b (Ω)	R_{ct} (Ω)	D_{Li+} (cm^2 s^{-1})
LZTO	5.461	223.3	1.06×10^{-15}
LZTO/G	5.229	187.6	3.65×10^{-15}

References

S1 W.J.H. Borghols, M. Wagemaker, U. Lafont, E.M. Kelde, F.M. Mulder, Size effects in the $Li_{4+x}Ti_5O_{12}$ spinel, J. Am. Chem. Soc. 131 (2009) 17786-17792.

S2 H. Ge, N. Li, D. Li, C. Dai, D. Wang, Electrochemical characteristics of spinel $Li_4Ti_5O_{12}$ discharged to 0.01 V, Electrochem. Commun. 10 (2008) 719-722.

S3 Z.S. Hong, X.Z. Zheng, X.K. Ding, L.L. Jiang, M.D. Wei, K.M. Wei, Complex spinel titanate nanowires for a high rate lithiumion battery, Energy Environ. Sci. 4 (2011) 1886-1891.

S4 L.J. Wang, B.K. Chen, Z.H. Meng, B.M. Luo, X.J. Wang, Y.Y. Zhao, High performance carbon-coated lithium zinc titanate as an anode material for lithium-ion batteries, Electrochim. Acta 188 (2016) 135-144.

S5 W. Chen, H.F. Liang, W.J. Ren, L.Y. Shao, J. Shu, Z.C. Wang, Complex spinel titanate as an advanced anode material for rechargeable lithium-ion batteries, J. Alloys Compd. 611 (2014) 65-73.

S6 H.Q. Tang, Q. Weng, Z.Y. Tang, Chitosan oligosaccharides: A novel and efficient water soluble binder for lithium zinc titanate anode in lithium-ion batteries, Electrochim. Acta 151 (2015) 27-34.

S7 Z.H. Meng, L.J. Wang, X.X. Li, G.Q. Zhang, H.Y. Li, Synthesis of high performance carbon-coated lithium zinc titanate via an EDTA-assisted route, Int. J. Hydrogen Energy 42 (2017) 2177-2186.

S8 Z.H. Meng, X.F. Chen, L.J. Wang, F. Wang, Synthesis of high rate capability N-doped carbon coated on lithium zinc titanate via a surfactant-assisted solid-state route,

RSC Adv.7 (2017) 54258-54265.

S9 H.Q. Tang, J.T. Zhu, C.X. Ma, Z.Y. Tang, Lithium cobalt oxide coated lithium zinc titanate anode material with an enhanced high rate capability and long lifespan for lithium-ion batteries, Electrochim. Acta 144 (2014) 76-84.

S10 H.Q. Tang, L.L. Zan, J.T. Zhu, Y.H. Ma, N.Q. Zhao, Z.Y. Tang, High rate capacity nanocomposite lanthanum oxide coated lithium zinc titanate anode for rechargeable lithium-ion battery, J.Alloys Compd. 667 (2016) 82-90.

S11 T. Liu, H.Q. Tang, L.L. Zan, Z.Y. Tang, Comparative study of $Li_2ZnTi_3O_8$ anode material with good high rate capacities prepared by solid state, molten salt and sol–gel methods, J. Electroanal. Chem. 771 (2016) 10-16.

S12 C. Chen, C.C. Ai, X.Y. Liu, Y.X. Wu, Advanced electrochemical properties of Ce-modified $Li_2ZnTi_3O_8$ anode material for lithium-ion batteries, Electrochim. Acta 227 (2017) 285-293.

S13 Z.F. Li, H. Li, Y.H. Cui, Z.G. Du, Y.H. Ma, C.X. Ma, Z.Y. Tang, Li_2MoO_4 modified $Li_2ZnTi_3O_8$ as a high property anode material for lithium ion battery, J. Alloys Compd. 692 (2017) 131-139.

S14 H.Q. Tang, Y.K. Zhou, L.L. Zan, N.Q. Zhao, Z.Y. Tang, Long cycle life of carbon coated lithium zinc titanate using copper as conductive additive for lithium ion batteries, Electrochim. Acta 191 (2016) 887-894.

S15 B.K. Chen, C.J. Du, Y.Z. Zhang, R.X. Sun, L. Zhou, L.J. Wang, A new strategy for synthesis of lithium zinc titanate as an anode material for lithium ion batteries, Electrochim. Acta 159 (2015) 102-110.
